# Diagnostic metabolite biomarkers of chronic typhoid carriage

**DOI:** 10.1371/journal.pntd.0006215

**Published:** 2018-01-26

**Authors:** Elin Näsström, Pär Jonsson, Anders Johansson, Sabina Dongol, Abhilasha Karkey, Buddha Basnyat, Nga Tran Vu Thieu, Tan Trinh Van, Guy E. Thwaites, Henrik Antti, Stephen Baker

**Affiliations:** 1 Department of Chemistry, Umeå University, Umeå, Sweden; 2 Department of Clinical Microbiology and the Laboratory for Molecular Infection Medicine Sweden, Umeå University, Umeå, Sweden; 3 Oxford University Clinical Research Unit, Patan Academy of Health Sciences, Kathmandu, Nepal; 4 The Hospital for Tropical Diseases, Wellcome Trust Major Overseas Programme, Oxford University Clinical Research Unit, Ho Chi Minh City, Vietnam; 5 Centre for Tropical Medicine and Global Health, Oxford University, Oxford, United Kingdom; 6 The Department of Medicine, The University of Cambridge, Cambridge, United Kingdom; Massachusetts General Hospital, UNITED STATES

## Abstract

**Background:**

*Salmonella* Typhi and *Salmonella* Paratyphi A are the agents of enteric (typhoid) fever; both can establish chronic carriage in the gallbladder. Chronic *Salmonella* carriers are typically asymptomatic, intermittently shedding bacteria in the feces, and contributing to disease transmission. Detecting chronic carriers is of public health relevance in areas where enteric fever is endemic, but there are no routinely used methods for prospectively identifying those carrying *Salmonella* in their gallbladder.

**Methodology/Principal findings:**

Here we aimed to identify biomarkers of *Salmonella* carriage using metabolite profiling. We performed metabolite profiling on plasma from Nepali patients undergoing cholecystectomy with confirmed *S*. Typhi or *S*. Paratyphi A gallbladder carriage (and non-carriage controls) using two-dimensional gas chromatography coupled with time-of-flight mass spectrometry (GCxGC-TOFMS) and supervised pattern recognition modeling. We were able to significantly discriminate *Salmonella* carriage samples from non-carriage control samples. We were also able to detect differential signatures between *S*. Typhi and *S*. Paratyphi A carriers. We additionally compared carriage metabolite profiles with profiles generated during acute infection; these data revealed substantial heterogeneity between metabolites associated with acute enteric fever and chronic carriage. Lastly, we found that *Salmonella* carriers could be significantly distinguished from non-carriage controls using only five metabolites, indicating the potential of these metabolites as diagnostic markers for detecting chronic *Salmonella* carriers.

**Conclusions/Significance:**

Our novel approach has highlighted the potential of using metabolomics to search for diagnostic markers of chronic *Salmonella* carriage. We suggest further epidemiological investigations of these potential biomarkers in alternative endemic enteric fever settings.

## Author summary

Enteric fever, caused by typhoidal *Salmonella* serovars, remains a substantial public health problem in many low- and middle-income countries. The human-restricted nature of these organisms combined with the development of new vaccines suggests that regional elimination of enteric fever should be possible. However, individuals that chronically carry *Salmonella* in their gallbladder, such as the notorious Typhoid Mary, complicates enteric fever transmission and maintain circulation of the organisms. The prospective detection of chronic *Salmonella* carriers is therefore a critical step for regional enteric fever elimination. However, there are currently no diagnostic methods routinely in use for this purpose. Here, we used a novel method for identifying chronic *Salmonella* carriers by comparing metabolite patterns in plasma samples from patients with chronic *Salmonella* carriage against non-carriage controls. We could significantly distinguish *Salmonella* carriers from non-carriers based on a large set of metabolites. Five metabolites were then highlighted, after comparing metabolite patterns obtained during chronic *Salmonella* carriage and acute enteric fever respectively, which could significantly distinguish *Salmonella* carriers from non-carriers. These potential biomarkers require further evaluation in epidemiological investigations of enteric fever in alternative endemic settings but this study provides a first step towards improved detection of *Salmonella* carriers.

## Introduction

Enteric fever is a systemic infection caused primarily by *Salmonella enterica* serovars Typhi (*S*. Typhi) and Paratyphi A (*S*. Paratyphi A); the disease has the highest incidences in low- and middle-income countries with limited access to clean water and poor hygiene standards [1]. The causative agents are transmitted via the fecal-oral route and, after entering the gastrointestinal tract, they translocate through gut mucosa and spread systemically, reaching the liver, spleen, bone marrow, and gallbladder [2]. There are limited data on the precise modes of pathogenesis and interactions between invasive *Salmonella* and the human host, but the organisms are thought to employ several strategies for avoiding immune defenses [3]. One of the principal covert mechanisms of *S*. Typhi and *S*. Paratyphi A is the ability to colonize the gallbladder, resist the antimicrobial activity of bile [4], and induce a chronic carriage [5,6]. Colonization of the gallbladder is thought to be facilitated by the formation of biofilms [7], which may be associated with an inability to effectively treat carriage with antimicrobials [5].

It is estimated that approximately 2–5% of individuals living in endemic areas that have experienced an episode of enteric fever will become a chronic carrier [8]. Typically, chronic carriers, such as Typhoid Mary, are asymptomatic [9] and many have no history of an acute disease episode [10], making the prospective detection of *S*. Typhi or *S*. Paratyphi A carriers a major challenge. Nonetheless, intermittent fecal shedding of bacteria is thought to be a major contributing factor for disease maintenance in endemic areas and the detection of these individuals is considered to be a public health priority for reducing the disease burden [11,12]. Current methods for the prospective detection of *Salmonella* chronic carriers include serial fecal culture[13], the detection of the bacteria in bile or on gallstones after cholecystectomy[5], and elevated antibody responses against the Vi polysaccharide antigen[14]. However, all these methods have various limitations (e.g. logistics, invasiveness, and sensitivity) and are seldom performed [5,13,15].

New approaches for detecting *Salmonella* carriers are warranted, and we have previously used metabolomics for identifying biomarkers associated with acute enteric fever [16,17]. Here, we aimed to identify metabolite biomarkers specific for *S*. Typhi or *S*. Paratyphi A carriage. Using plasma samples from patients undergoing cholecystectomy with confirmed *S*. Typhi or *S*. Paratyphi A gallbladder carriage and appropriate controls we performed two-dimensional gas chromatography coupled with time-of-flight mass spectrometry (GCxGC-TOFMS). After generating metabolite profiles, our primary strategy was to use chemometric bioinformatics to investigate if carriers could be differentiated from non-carriers, thus generating a biomarker pattern (latent biomarker) of carriage that may be developed into a diagnostic methodology. As opposed to focusing on single metabolites as diagnostic markers, we hypothesize that a combination of co-varying metabolites could potentially provide a means for more sensitive and specific biomarkers of typhoid carriage. Additionally, as secondary aims we sought to distinguish between *S*. Typhi and *S*. Paratyphi A carriers and to investigate if the identified metabolite profiles were unique to carriage with respect to those identified during acute infection [16].

## Methods

### Ethics statement

This study was conducted according to the principles expressed in the Declaration of Helsinki and was approved by the institutional ethical review boards of Patan Hospital, The Nepal Health Research Council, and the Oxford Tropical Research Ethics Committee (OXTREC, Reference number: 2108). All enrollees were required to provide written informed consent for the collection, use and storage of tissue and blood collected surgery.

### Study site population and study design

The study was conducted at Patan Hospital, a 318-bed government hospital located in the Lalitpur Sub-Metropolitan City in the Kathmandu valley, Nepal. Non-specific febrile disease is common at Patan Hospital and *S*. Typhi and *S*. Paratyphi A are the most common bacteria cultured from blood of febrile patients in this location [18]. The study generating the plasma samples has for this investigation been previously described [19]. Briefly, EDTA blood was collected from a subset of patients undergoing cholecystectomy at Patan Hospital from June 2007 to October 2010. A questionnaire related to the patient’s health and demographics was administered prior to surgery along with a stool sample for microbiological culture. Surgeons collected bile samples and gallbladder tissue during the procedure. After recruiting 1,377 cholecystectomy patients over three years and culturing their bile we identified 24 and 22 individuals with *S*. Typhi and *S*. Paratyphi A inside their gallbladder, respectively; 35/46 (76%) were female and the median age was 34.5 years (range; 20–67).

### Plasma samples

Plasma samples were stored at -80°C until analysis. The samples constituted a subset of those enrolled in the study and were comprised of plasma samples from patients with confirmed *S*. Typhi (n = 12) and *S*. Paratyphi A (n = 5) gallbladder carriage i.e. individuals from whom *S*. Typhi or *S*. Paratyphi A was isolated from their bile after cholecystectomy. We additionally analyzed plasma from individuals who underwent surgery but had sterile bile (n = 20) i.e. a surgical control population without exhibiting growth of bacteria in their bile. Additional patient information and patient group metadata can be found in S1 File and S1 Table.

### Sample preparation for metabolomics analysis

For extraction and derivatization of the samples prior to analysis with GCxGC-TOFMS we used the plasma protocol for metabolomics analysis at the Swedish Metabolomics Centre (SMC) with a 50μl starting volume [20] (and described in detail in [16]). Quality control (QC) samples were prepared by pooling 100μl aliquots of seven samples from each class.

### GCxGC-TOFMS analysis

The extracted and derivatized plasma samples were analyzed on a GCxGC-TOFMS as described previously [16] but with minor modifications. The MS transfer line was set at 325°C and the detector voltage at 1,700V. The analytical run order was constructed by randomizing the three sample groups (*S*. Typhi carriers, *S*. Paratyphi A carriers, and non-carriage controls). The run also included QC samples incorporated at the beginning and end of the run and after every sixth sample. In addition, blank samples (milli-Q water and extraction mix) and n-alkane series (C8-C40) for retention index calculation were analyzed.

### Data processing and metabolite identification

The GCxGC-TOFMS data was processed as NetCDF files using an in-house Matlab script (MATLAB R2014b, Mathworks, Natick, MA, USA) applying hierarchical multivariate curve resolution (HMCR) [21] on GCxGC-TOFMS data. The processing resulted in resolved chromatographic peaks with semi-quantitative metabolite concentrations and corresponding mass spectra. The mass spectra were subjected to library search in NIST MS Search 2.0 to give the peaks a putative annotation. In-house libraries from SMC and publicly available libraries (from US National Institute of Science and Technology (NIST) and the Max Planck Institute in Golm (http://gmd.mpimp-golm.mpg.de/)) together with libraries of all sample peaks (to detect split peaks) and peaks from the acute enteric fever study were used in the identification. Split peaks and metabolites with a different number of TMS groups were investigated by comparing retention indexes, mass spectral matches, raw data profiles and loading positions in a PCA model and for peaks with comparable values/profiles only one peak was included in further analysis. Other criteria for peak exclusion were: low quality peak/mass spectrum, column bleed artifacts, internal standard, high analytical run order correlation (Pearson correlation coefficient >❘0.5❘), and highly deviating QC samples (RSD>0.5, peaks with RSD between 0.3 and 0.5 were manually investigated).

### Pattern recognition/multivariate data analysis

Multivariate projection methods provide a statistical tool for deciphering multivariate or latent biomarkers based on combinations of variables (e.g. metabolites, proteins etc.). Initially unsupervised modeling with principal component analysis (PCA) [22] was used for peak investigation during the identification process, and for data overview with metabolites selected for inclusion. From PCA modeling the general trends in the metabolite data can be obtained with possible outliers. The variable raw data was investigated and highly deviating samples were detected in a few metabolites. Metabolites with one sample having a centered and UV scaled value >4 in a PCA model with all samples were investigated. PCA models with and without missing value replacement were obtained to investigate the effect. In general, metabolites with a value >5 were selected for missing value replacement but also metabolites where the deviating sample was affecting the significance of the metabolite (either by reinforcing a weak trend or creating an opposite trend compared to the majority of the samples in the same class). All the following models were calculated with missing value replaced data. Unsupervised modeling was followed by supervised modeling using orthogonal partial least squares-discriminant analysis (OPLS-DA) [23]. OPLS-DA models were obtained to investigate the metabolite profiles related to chronic carriage for i) a three-class model with *S*. Typhi carriers, *S*. Paratyphi A carriers, and non-carriage controls, ii) a combination of *S*. Typhi and *S*. Paratyphi A carriage samples compared to non-carriage controls, iii) separate models for *S*. Typhi and *S*. Paratyphi A carriage samples compared to non-carriage controls and iv) for *S*. Typhi carriage samples compared to *S*. Paratyphi A carriage samples. All data was centered and scaled to unit variance and all OPLS-DA models were validated with a seven-fold cross-validation [24]. The multivariate significance criterion was based on the latent significance concept recently developed in our research group (Jonsson et al., submitted). The latent significance concept takes advantage of the unique features of OPLS modeling to highlight significant metabolites. In OPLS, the variation in the measured variables (here metabolite concentrations) can be divided into one part related to the response of interest (here class information regarding carriage) and into one part unrelated to the response. In the latent significance concept the orthogonal variation is subtracted, creating latent model covariance loadings, w_latent_, to be able to highlight metabolites with a significant alteration only focusing on the response of interest. Univariate *p*-values were calculated using the Mann-Whitney U-test and metabolites with *p*≤0.05 were considered univariate significant. The selection of significant metabolites was based on multivariate significance only. To investigate how well metabolite patterns and the individual metabolites could distinguish between the sample groups receiver operating characteristic (ROC) curves were constructed. For the metabolite panels the cross-validated scores from the OPLS-DA models were used to construct the ROC curves and for the individual metabolites the relative metabolite concentrations were used. Area under the curve (AUC) values were calculated from the ROC curves with values ranging from 0.5 to 1 (where 0.5 represents a random classifier and 1 represents a perfect classifier). The bootstrap percentile resampling method (using 1,000 bootstrappings) was used to calculate 95% confidence intervals for the AUC values[25]. Modeling was performed in SIMCA (version 14, Umetrics, Umeå, Sweden), ROC curve analysis was performed in Matlab (R2014b, Mathworks, Natick, MA, USA) and figures were created in GraphPad Prism (5.04; GraphPad Software Inc., La Jolla, CA, USA).

### Comparison of metabolites

In order to compare the profiles during acute enteric fever and chronic carriage the metabolites found to be significant for separating carriers from non-carriers in the current study were compared to significant metabolites separating acute enteric fever samples from afebrile controls in a previous study [16]. Initially, the mass spectra for the carriage samples were compared to the acute enteric fever samples during the identification process to find matches between both putatively identified and unidentified metabolites. The metabolites were then compared by significance and direction of change to highlight both similarities and differences between acute enteric fever and chronic carriage.

## Results

### Plasma metabolites in *Salmonella* carriers

Plasma samples from patients with confirmed *S*. Typhi (n = 12) and *S*. Paratyphi A (n = 5) gallbladder carriage together with control samples without gallbladder carriage of any bacteria (n = 20) were analyzed by GCxGC-TOFMS (S1 File and S1 Table). This resulted in 691 detected peaks and after further investigation 195 putative metabolites were selected for downstream analysis. Exclusion was mainly associated with split peaks, low quality peaks or mass spectra, peaks with a high correlation with run order, and deviation in quality control samples. Of the selected peaks 69/195 (35.4%) had a putative annotation, 8/195 (4.1%) had an assigned metabolite class, 18/195 (9.2%) were of uncertain identity, and 100/195 (51.3%) were of unknown identity (S2 Table). Examining the raw processed data by principal component analysis (PCA) further revealed ten putative metabolites that had extremely high concentration levels in single samples, these metabolite concentrations were replaced by missing values to avoid misclassification.

### Metabolite profiles distinguishing *Salmonella* carriers from non-carriers

**Fig 1:**
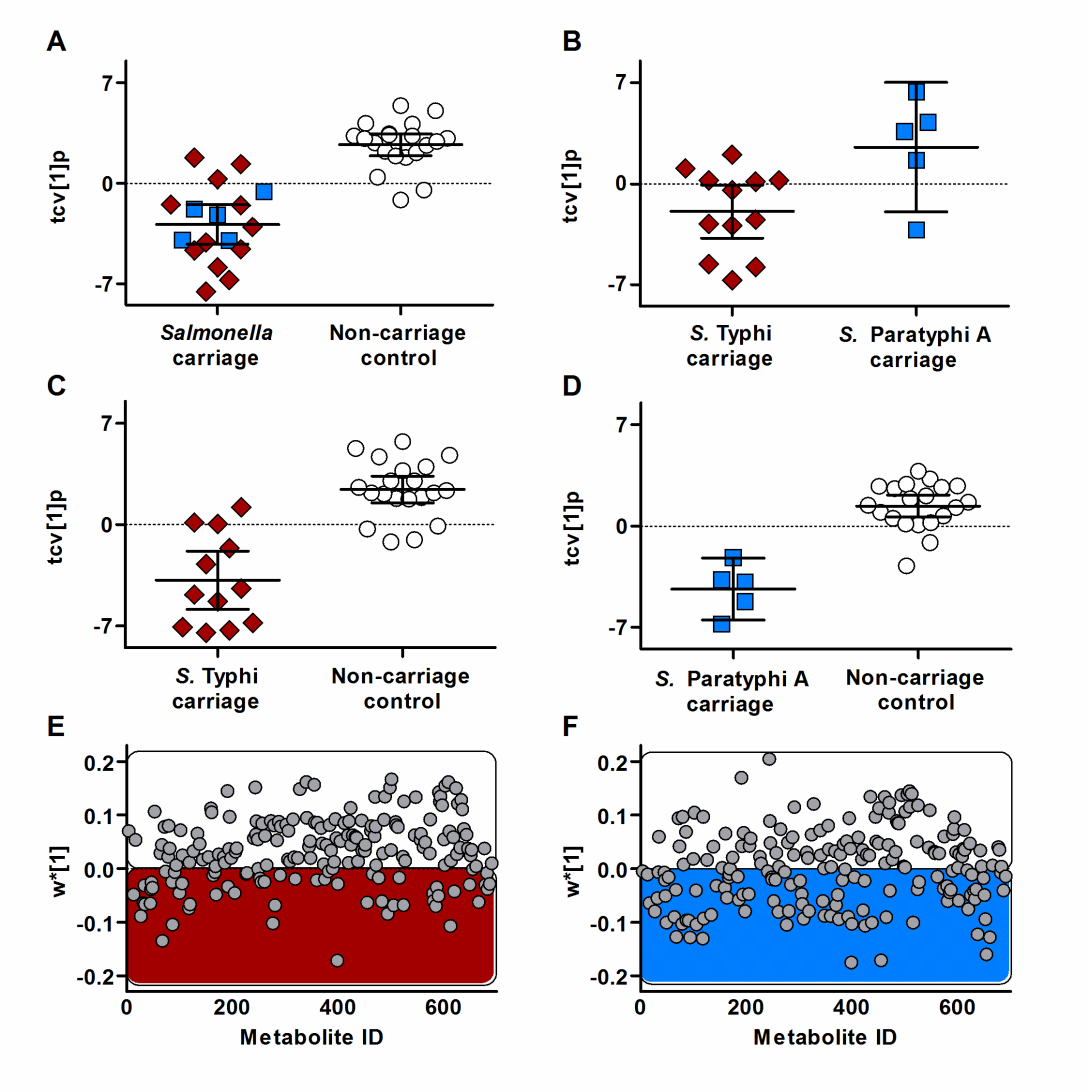
Pairwise OPLS-DA models of *Salmonella* carriage and non-carriage control samples. Models based on 195 metabolites generated from GCxGC-TOFMS analysis of plasma samples from patients in Nepal undergoing cholecystectomy. Panels A-D are showing cross-validated scores for the first predictive component (tcv[1]p) in the respective OPLS-DA model (error bars: mean scores with 95% confidence intervals). *S*. Typhi carriage (n = 12), *S*. Paratyphi A carriage (n = 5), and non-carriage controls (n = 20). (A) *Salmonella* carriage samples significantly separated from non-carriage controls (*p* = 2.8*10^−6^). (B) *S*. Typhi carriage samples separated from *S*. Paratyphi A carriage samples (*p* = 0.070). (C) *S*. Typhi carriage samples significantly separated from non-carriage controls (*p* = 3.1*10^−6^). (D) *S*. Paratyphi A carriage samples significantly separated from non-carriage controls (*p* = 3.7*10^−4^). Panels E-F are showing the distribution of the 195 metabolites using model covariance loadings for the first predictive component (w*[1]) in the respective OPLS-DA model. (E) More metabolites shifted towards higher relative concentration in non-carriage controls compared to *S*. Typhi carriage samples. (F) Metabolites more equally distributed between the *S*. Paratyphi A carriage samples and the non-carriage controls. Additional model information is shown in Table 1.

To identify metabolite profiles that may be associated with *Salmonella* carriage we used unsupervised multivariate modeling and generated a primary PCA model of the 195 metabolites in the 37 plasma samples. The model indicated a direction of separation of the non-carriage controls from the *S*. Typhi and *S*. Paratyphi A carriage samples; this separation was chiefly along the second principal component (S1A Fig and Table 1). We next applied supervised modeling through OPLS-DA to further investigate the strength and details of the trend observed in the PCA model. A three-class OPLS-DA model of *S*. Typhi carriers, *S*. Paratyphi A carriers, and non-carriage controls was fitted to generate an overview of the relation between the sample classes. The plasma samples from the *Salmonella* carriers were clearly segregated from the non-carriage samples along the first component whilst the *S*. Paratyphi A carriage samples were separated from the *S*. Typhi carriage samples along the second component (*p* = 0.0031) (S1B Fig and Table 1). For a more detailed investigation of the metabolite profiles separating the carriage samples from the non-carriage samples a two-class OPLS-DA model was generated with *S*. Typhi and *S*. Paratyphi A carriage samples combined in one class and non-carriage controls in the other class. The metabolite profiles from the *Salmonella* carriage samples were significantly divergent from the profiles of the non-carriage samples (*p* = 2.8*10^−6^) (Fig 1A and Table 1).

**Table 1:** Multivariate model overview.

**Model**^a^	**Num. met.**^b^	**Comp.**^c^	**R**^**2**^**X**^d^	**R**^**2**^**Y**^d^	**Q**^2^^d^	**CV-ANOVA**^e^	**AUC (95% CI)** **CV-scores**^f^
PCA samples, before missing rep.	195	4	0.507	-	0.217	-	-
PCA samples, after missing rep.	195	4	0.518	-	0.243	-	-
3 classes (*S*. Typhi carriers vs. *S*. Paratyphi A carriers vs. non-carriage controls)	195	2+2	0.475	0.817	0.509	0.0031	-
*Salmonella* carriage vs. non-carriage controls	195	1+1	0.323	0.774	0.613	2.8*10^−6^	0.974 (0.921–1)
*S*. Typhi carriage vs. non-carriage controls	195	1+1	0.345	0.781	0.607	3.1*10^−5^	0.950 (0.864–1)
*S*. Paratyphi A carriage vs. non-carriage controls	195	1+1	0.234	0.921	0.630	3.7*10^−4^	0.990 (0.940–1)
*S*. Typhi carriage vs. *S*. Paratyphi A carriage	195	1+0	0.198	0.692	0.321	0.070	0.833 (0.524–1)
*Salmonella* carriage vs. non-carrier controls	5	1+0	0.417	0.635	0.604	1.4*10^−7^	0.935 (0.836–1)

^a^ All models are two-class OPLS-DA models unless stated otherwise.

^b^ Num. met.: The number of metabolites the model is based on.

^c^ Comp: The number of predictive model components followed by the number of orthogonal model components.

^d^ R^2^X: The amount of variation in X explained by the model, R^2^Y: The amount of variation in Y explained by the model, Q^2^: The amount of variation in Y predicted by the model.

^e^ CV-ANOVA: p-value based on cross-validated data showing the significance of the model.

^f^ AUC (95% CI) CV-scores: Area under the curve (AUC) values for receiver operating characteristic (ROC) curves based on cross-validated scores (tcv) from the OPLS-DA models. AUC values ranging between 0.5 and 1. 95% confidence intervals based on 1000 bootstrappings are given within parenthesis.

### Differentiating metabolites between *S*. Typhi and *S*. Paratyphi A carriers

We next segregated metabolite profiles generated in the *Salmonella* carriage plasma samples by infecting organism and performed further pairwise OPLS-DA comparisons with the non-carriage controls. The models revealed that the *S*. Typhi carriage samples and the *S*. Paratyphi A carriage samples were clearly segregated from the non-carriage controls (*p* = 3.1*10^−5^ and *p* = 3.7*10^−4^, respectively) (Fig 1C and 1D and Table 1), as indicated in the combined *S*. Typhi and *S*. Paratyphi A model. Further investigation of the metabolite distributions (using the model covariance loadings for the first OPLS-DA component w*[1]) highlighted a shift of metabolites towards the non-carriage control group in the *S*. Typhi model (Fig 1E) (i.e. more metabolites with a higher relative concentration in the non-carriage group than in the *S*. Typhi group). Notably, this pattern was not observed in the *S*. Paratyphi A carriage model, in which the metabolites were more evenly distributed, although the *S*. Paratyphi A group was comprised of few samples (Fig 1F). The three-class OPLS-DA model, together with the different appearance of the model covariance loadings of the *S*. Typhi and *S*. Paratyphi A models, indicated potential differences between metabolite profiles in the *S*. Typhi carriage samples and *S*. Paratyphi A carriage samples. Therefore, we generated an OPLS-DA model comparing the metabolite profiles between the *S*. Typhi and *S*. Paratyphi A carriers. This analysis resulted in a weaker model for the identification of differences in metabolite profiles between the two *Salmonella* serovars during carriage (*p* = 0.07) (Fig 1B and Table 1).

### Further investigation of metabolite patterns during *Salmonella* carriage

**Fig 2:**
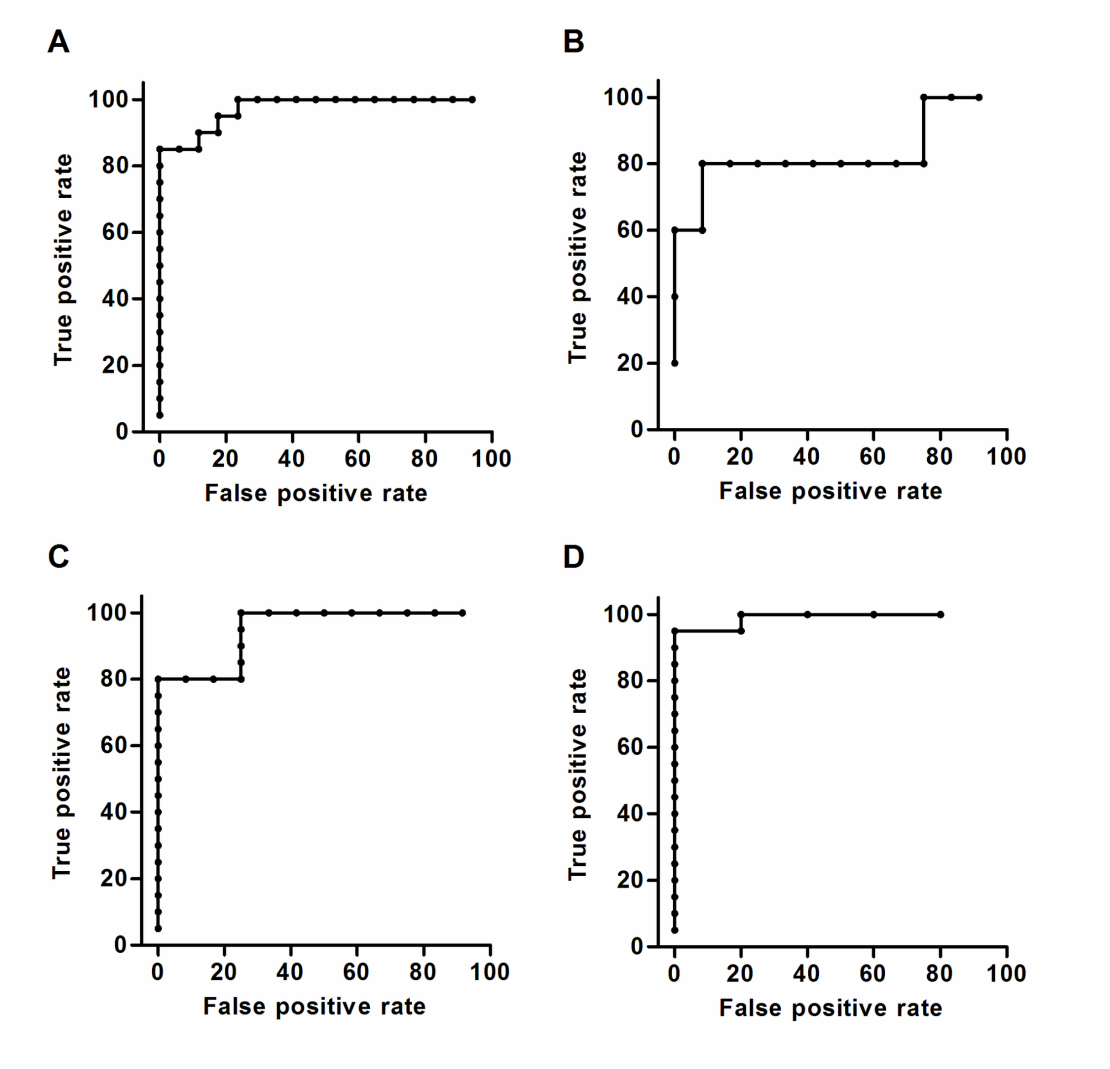
ROC curves for metabolite patterns in OPLS-DA models of *Salmonella* carriage and non-carriage control samples. Panels A-D are showing ROC curves with false positive rates (i.e. 1-specificity) and true positive rates (i.e. sensitivity) on the x- and y-axes respectively. The ROC curves are constructed from cross-validated scores (tcv) from pairwise OPLS-DA models based on 195 metabolites. AUC values are presented along with 95% confidence intervals. (A) *Salmonella* carriage samples compared to non-carriage controls, AUC = 0.974 (0.921–1). (B) *S*. Typhi carriage samples compared to *S*. Paratyphi A carriage samples, AUC = 0.833 (0.524–1). (C) *S*. Typhi carriage samples compared to non-carriage controls, AUC = 0.950 (0.864–1). (D) *S*. Paratyphi A carriage samples compared to non-carriage controls, AUC = 0.990 (0.940–1).

To further investigate the diagnostic potential of the metabolite patterns and the individual metabolites ROC curves were constructed. ROC curves for the metabolite patterns based on the cross-validated scores from the pairwise OPLS-DA models are shown in Fig 2. For all pairwise OPLS-DA models comparing *Salmonella* carriage samples and non-carriage controls (with *S*. Typhi and *S*. Paratyphi A carriage samples combined or separated) the ROC curves indicated strong diagnostic potential with AUC values ≥ 0.95 (Fig 2A–2C). The weaker OPLS-DA model obtained when comparing *S*. Typhi and *S*. Paratyphi A carriage samples resulted in a ROC curve with an AUC value of 0.833 (Fig 2D). Focusing only on the comparison between the combined *Salmonella* carriage samples and the non-carriage controls ROC curves were constructed for each of the 195 metabolites (included in the OPLS-DA model) based on the relative metabolite concentrations. AUC values from these ROC curves are listed in S2 Table. None of the individual metabolites had an AUC value as high as or higher than the AUC value for the metabolite pattern (Fig 2A).

### Comparison of acute enteric fever and chronic carriage

**Fig 3:**
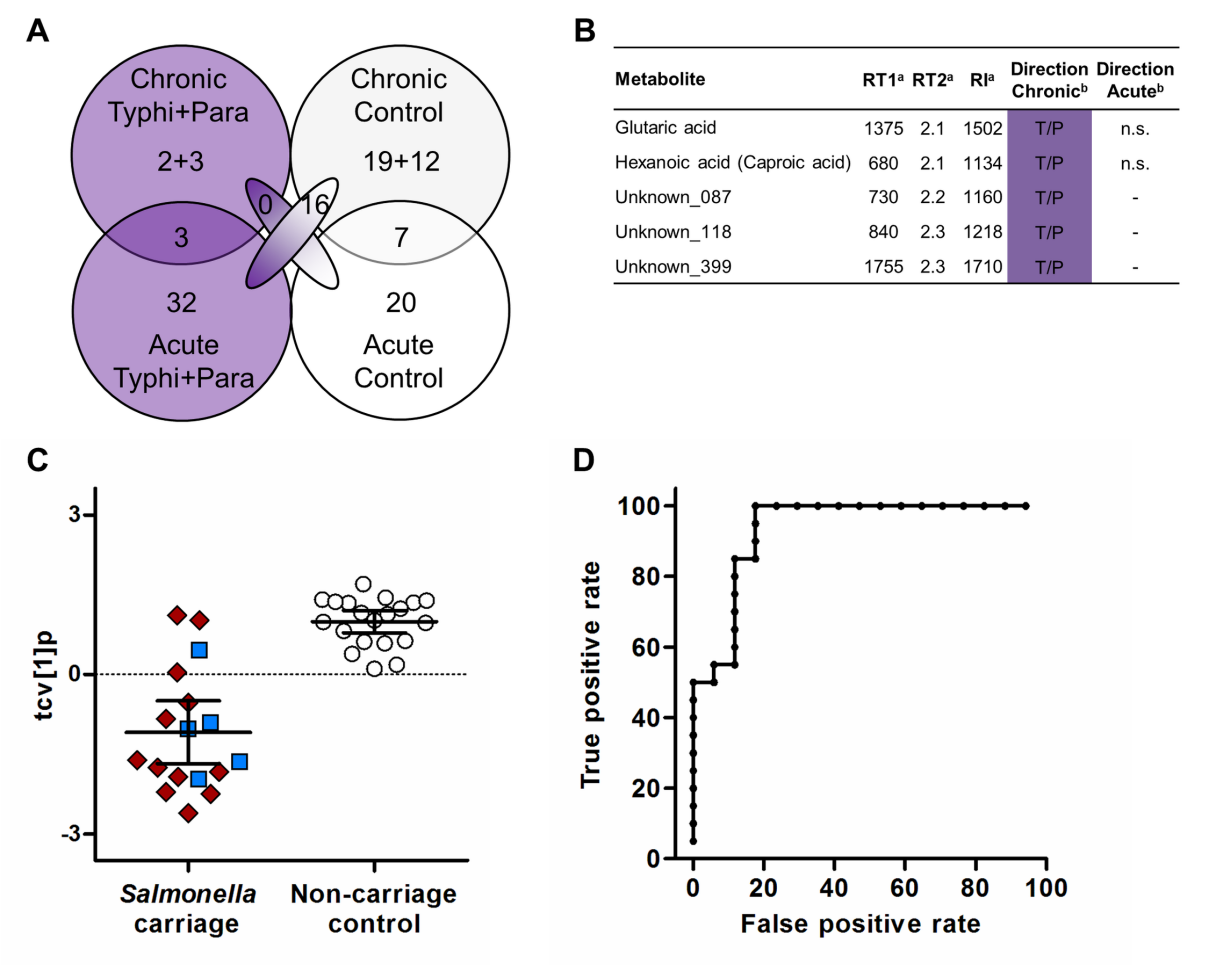
Comparison of metabolites between acute enteric fever and chronic carriage. (A) Venn diagram of metabolites significant in OPLS-DA models separating *Salmonella* carriage samples from non-carriage controls and patients with acute *S*. Typhi or *S*. Paratyphi A infections from afebrile controls. Metabolites with the same direction of change in chronic and acute infection are shown in the overlapping circles while metabolites with different direction are shown in the overlapping ellipses and metabolites only significant in one infection stage are shown in the reminder part of the circle. The numbers after the plus sign represent metabolites that are not present in the acute infection dataset. (B) Table of metabolites only significant in chronic infection having a higher relative concentration in the *S*. Typhi/*S*. Paratyphi A carriage group compared to non-carriage controls. ^a^ RT1: 1st dim. retention time (s), RT2: 2nd dim. retention time (s) and RI: retention index. ^b^ Direction: direction of change in relative metabolite concentration; metabolites having higher relative concentration in the *S*. Typhi/*S*. Paratyphi A group marked with T/P, non-significant metabolites marked with n.s. and metabolites not present marked with -. (C) Cross-validated scores for the first predictive component (tcv[1]p) in an OPLS-DA model based on the five metabolites highlighted in B and showing the separation of *Salmonella* carriage samples (*S*. Typhi carriage–n = 12 and *S*. Paratyphi A carriage–n = 5) from non-carriage controls (n = 20) but with overlap of four *Salmonella* carriage samples (*p* = 1.4*10^−7^). Error bars represent mean score values with 95% confidence intervals. Additional model information is shown in Table 1. (D) ROC curve with false positive rates (i.e. 1-specificity) and true positive rates (i.e. sensitivity) on the x- and y-axes respectively. The ROC curve was constructed with cross-validated scores (tcv) from the pairwise OPLS-DA model comparing *Salmonella* carriage samples and non-carriage controls based on five metabolites. AUC value with 95% confidence interval: 0.935 (0.836–1).

In a prior study we investigated metabolites associated with acute enteric fever in patients with culture-confirmed acute *S*. Typhi infections, *S*. Paratyphi A infections, and afebrile controls [16]. Using these existing data, we compared the metabolite profiles related to acute enteric fever and chronic carriage; both conducted in the same setting in Nepal. As our aim was to segregate *Salmonella* carriers from non-carriers, we used models where *S*. Typhi and *S*. Paratyphi A infections were combined and compared to controls (non-carriage controls from the current investigation and afebrile controls from the former investigation) to identify significant metabolites differentiating between the sample groups. The resulting metabolite comparison is summarized in Fig 3A (S3 Table). Investigating comparable metabolites between acute enteric fever and chronic carriage highlighted three metabolites that were increased in the *S*. Typhi/*S*. Paratyphi A group; seven were increased in the control group. However, there were more metabolites with different directions of change in acute infection and carriage with 16 metabolites increased in the *S*. Typhi/*S*. Paratyphi A group in acute infection. Notably, we observed a substantial number of metabolites that were significantly different in only acute infection or carriage respectively. Of particular interest were five metabolites (glutaric acid, hexanoic acid, and three metabolites with unknown identity) that were elevated in the *S*. Typhi/*S*. Paratyphi A group in the carriage samples only (Fig 3B). These metabolites were potentially differential and may have utility for prospectively identifying *Salmonella* carriers. Using these five metabolites to calculate an OPLS-DA model for *Salmonella* carriage in comparison to the non-carriage samples resulted in a significant separation between the groups (*p* = 1.4*10^−7^) (Fig 3C and Table 1). A ROC curve constructed using the cross-validated scores from this five metabolite OPLS-DA model indicated that the strong diagnostic potential for distinguishing between *Salmonella* carriers and non-carriage controls was maintained using only these five metabolites (Fig 3D). This ROC curve had an AUC value (0.935) that was higher than any of the AUC values from ROC curves of the five individual metabolites (S2 Fig and S2 Table).

## Discussion

Typhoid carriage remains one of greatest enigmas in infectious disease research. Due to the imperceptible nature of carriers the mechanisms and precise epidemiological role of *S*. Typhi carriage in humans are poorly defined. However, carriers are thought to be essential for disease maintenance and may be important for generating new genotypes. Therefore, prospectively detecting carriers is a key objective in regional enteric fever elimination. Here we employed metabolomics and chemometric bioinformatics to analyze plasma samples from patients in Nepal undergoing cholecystectomy that were confirmed carriers of *S*. Typhi or *S*. Paratyphi A. Using this patient population in an endemic enteric fever area we were able to generate metabolomic biomarker profiles indicative of *Salmonella* carriage. The descriptive nature of the study makes it difficult to reveal what the detected metabolite profiles physiologically reflect. However, notable patterns with diagnostic potential were identified and some of these aspects will be discussed.

By exploiting specific metabolite profiles we were able to separate *Salmonella* carriers from non-carriers in a multivariate model. Further, segregating the carriage group into *S*. Typhi carriers and *S*. Paratyphi A carriers and comparing them independently to non-carriage controls generated an equivalent separation of the two carriage groups from non-carriage controls. Notably, metabolite profiles, as appose to individual metabolites, showed greater diagnostic potential (in terms of AUC values for ROC curves). In addition, differences in the distribution of the metabolites between the two models were highlighted. We observed that the majority of the metabolites had a lower relative concentration in the *S*. Typhi carriage samples than the non-carriage controls. We are uncertain of the precise reason for a relative reduction in metabolite concentrations in the carriers compared to the controls, but speculate that this is evidence of *S*. Typhi controlling the inflammatory response. *S*. Typhi is a covert pathogen that has the ability to hide from the immune system and regulate inflammation during infection [26]. The Vi capsule polysaccharide, which protects outer membrane proteins from immunological interactions, likely controls much of this immune regulation [27,28]. During chronic carriage *S*. Typhi is within a protected, immune-privileged environment inside the gallbladder, and provoking an inflammatory response is incompatible with long-term persistence. Our data are consistent with *S*. Typhi manipulating systemic inflammatory responses during carriage, indicating that *S*. Typhi is perfectly adapted for long-term persistence in the gallbladder. Furthermore, *S*. Paratyphi A does not express the Vi polysaccharide and does not, therefore, regulate the inflammatory response in the same manner; the trend of increased metabolite concentrations in the non-carriage controls was not observed in the *S*. Paratyphi A model. Our data additionally indicated differences in metabolite profiles between *S*. Typhi and *S*. Paratyphi A carriers, but these differences were weaker than those observed between carriers and non-carriage controls. Although the key aim of this study was to differentiate carriers from non-carriers, distinguishing *S*. Typhi carriers from *S*. Paratyphi A carriers may be relevant in disease epidemiology and for studying the carriage mechanisms of these two organisms.

There is a need for improved diagnostic methods for acute enteric fever and chronic carriage; therefore we additionally compared the metabolite profiles between chronic carriers and acute enteric fever cases. We identified a greater number of differences than similarities in metabolite profiles between the acute infection and chronic carriage. Among the differences there were metabolites with a different direction of change in both conditions and also metabolites found significant in one of the two disease stages only. In identifying diagnostic biomarkers with the potential for prospectively detecting *S*. Typhi and *S*. Paratyphi A carriers, the most suitable candidates are metabolites with a higher concentration in carriers than non-carriers (more appropriate for point-of-care test development), and not relevant during acute infection. We identified five metabolites, including glutaric acid and hexanoic acid, which fulfilled these criteria. The profile of these five combined metabolites exhibited a stronger diagnostic potential in comparison to the five individual metabolites, highlighting the utility of using a combination of metabolite markers. We are uncertain of the relevance of these carboxylic acids, but these chemicals have antibacterial properties [29] and may be the result of altered gut microbiome substrate fermentation. Short-chain fatty acids, including hexanoic acid, are major fermentation products of the gut microbiome and concentrations can fluctuate by a shift in the composition of the gut microbiome [30–32]. Similarly, the same phenomenon can induce an increased catabolism of amino acids, resulting in an increase in the concentration of glutaric acid. It is speculative, but *Salmonella* spp. have been shown to alter the gut microbiome in murine models of infection [33,34]. The increase in hexanoic acid and glutaric acid observed during *Salmonella* carriage was not observed during acute enteric fever, suggesting that the release of organisms from the gallbladder into the duodenum may impact on the gastrointestinal microbiota. However, whether this discrepancy is a result of *Salmonella* associated alterations of the gut microbiome arising during the two disease stages or due to other metabolic processes remains unclear.

The major limitation of this study was the small sample size, which was difficult to overcome given the ethical, surgical, and diagnostic issues of identifying and confirming invasive *Salmonella* carriers. We suggest that resulting metabolite profiles should be validated in an additional, independent cohort. However, this was not possible in this setting due to the factors stated above. Furthermore, as all patients included in this study presented with gallbladder conditions we cannot rule out that the primary cause for the cholecystectomy may impact on the metabolite signatures. However, the surgical conditions were comparable for both carriers and non-carriers and, as there is a known association between *Salmonella* carriage and cholecystitis [35] there is significant value in investigating carriage in this patient cohort. The non-carrier controls included in this study were culture-negative, meaning that there was no growth of any bacteria in their bile. It would be of additional interest to include controls with gallbladder carriage of other bacteria in this comparison to be able to demonstrate that these metabolite are truly *Salmonella*-specific. Further studies in alternative endemic enteric fever populations are required to investigate the metabolite markers associated with *S*. Typhi and *S*. Paratyphi A carriage. If a set of metabolite markers for *Salmonella* carriage passes a further rigorous validation the next challenge is to convert this diagnostic panel into an accurate and inexpensive test suitable for use in resource-limited areas. An important feature of such a test is simultaneous detection of multiple biomarkers. A recent review summarizes current advantages in the field of multiplexed point-of-care testing[36]. In general, differing microfluidic techniques have great potential for such multiplexing, although many challenges remain. One promising example shows the measurement of three metabolites in human serum using microfluidic paper-based analytical devices[37].

In conclusion, our novel approach highlights the potential of using metabolomics to search for diagnostic markers of chronic *Salmonella* carriage. We identified metabolite patterns signifying carriage of *S*. Typhi and *S*. Paratyphi A in the gallbladder among a cohort of patients with cholelithiasis in Nepal. These findings are encouraging in the search for a diagnostic assay that may be able to access the reservoirs of *S*. Typhi and *S*. Paratyphi A carried asymptomatically within human populations.

## Supporting information

S1 Fig Unsupervised modeling and multi-class supervised modeling of *Salmonella* carriers and non-carriage controls. Scores for the two first components (t[1] and t[2]) in models based on 195 metabolites generated from GCxGC-TOFMS analysis of plasma samples from patients in Nepal undergoing cholecystectomy. Sample numbers: *S*. Typhi carriage–n = 12, *S*. Paratyphi A carriage–n = 5 and non-carriage control–n = 20. (A) PCA scores showing the distribution of the three sample groups, *S*. Typhi carriage, *S*. Paratyphi A carriage, and non-carriage controls with an indication of separation of non-carriage controls from the *Salmonella* carriage samples. (B) OPLS-DA scores showing the separation of non-carriage controls from the *Salmonella* carriage samples along the first component and the separation of *S*. Typhi carriage samples from the *S*. Paratyphi A carriage samples along the second component (*p* = 0.0031). Additional model information is shown in Table 1.
(TIF)

S2 Fig ROC curves for five individual metabolites significant during *Salmonella* carriage. Panels A-E are showing ROC curves with false positive rates (i.e. 1-specificity) and true positive rates (i.e. sensitivity) on the x- and y-axes respectively. The ROC curves are constructed from relative metabolite concentrations comparing *Salmonella* carriage samples to non-carriage controls. AUC values are presented along with 95% confidence intervals. (A) Hexanoic acid (Caproic acid), AUC = 0.841 (0.680–0.968). (B) Unknown_087, AUC = 0.697 (0.521–0.864). (C) Unknown_118, AUC = 0.756 (0.589–0.904). (D) Glutaric acid, AUC = 0.774 (0.565–0.887). (E) Unknown_399, AUC = 0.876 (0.726–1).
(TIF)

S1 File Patient information. (XLSX)

S1 Table Patient group metadata. (DOCX)

S2 Table Detected metabolites in plasma samples of *Salmonella* carriers analyzed with GCxGC-TOFMS. (DOCX)

S3 Table Comparison of metabolites between acute enteric fever and chronic carriage. (DOCX)
