# Supplementary figures and images for: Diagnostic metabolite biomarkers of chronic typhoid carriage
Source: PLoS Negl Trop Dis. 2018 Jan 26;12(1):e0006215. doi: 10.1371/journal.pntd.0006215 (PMC5802941; doi:10.1371/journal.pntd.0006215)

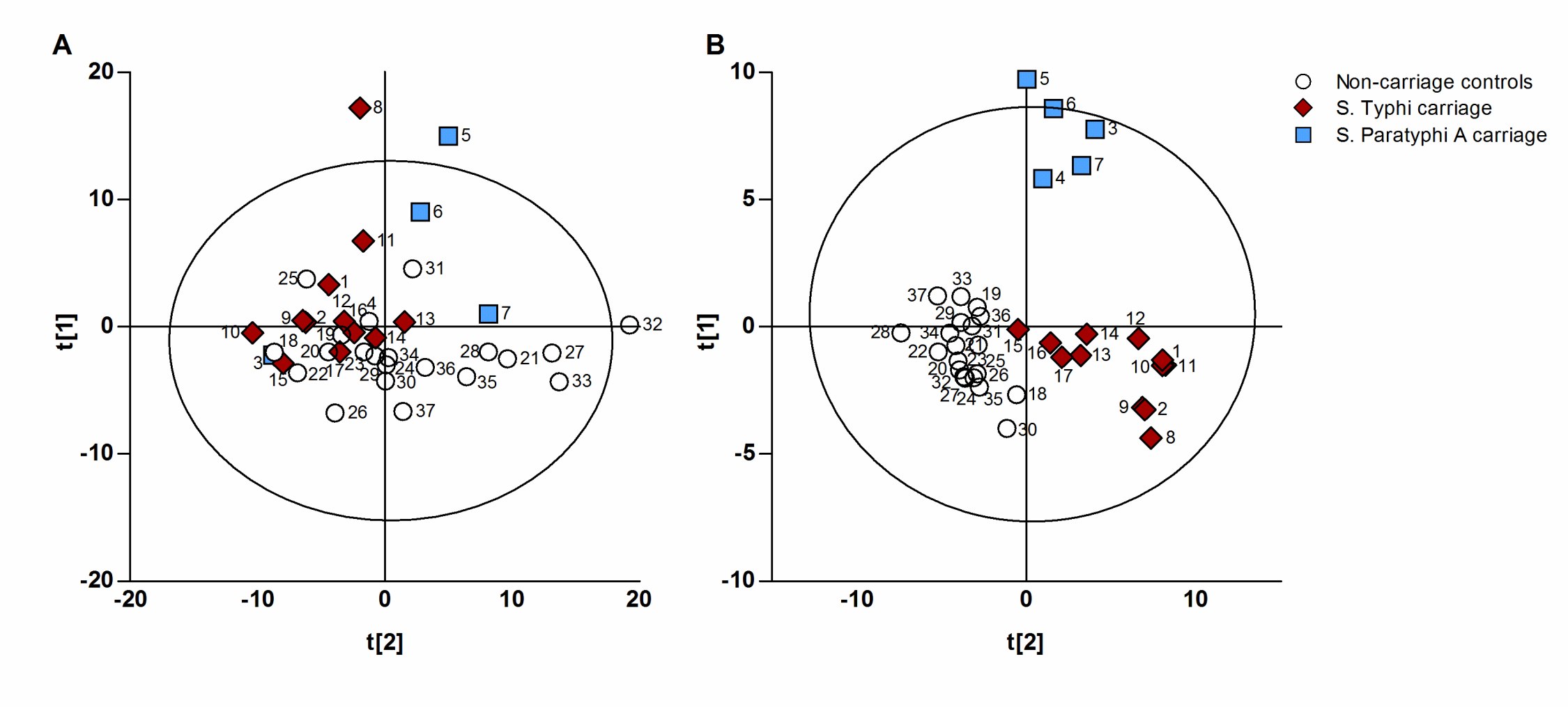

Supplement: S1 Fig — Scores for the two first components (t[1] and t[2]) in models based on 195 metabolites generated from GCxGC-TOFMS analysis of plasma samples from patients in Nepal undergoing cholecystectomy. Sample numbers: S. Typhi carriage–n = 12, S. Paratyphi A carriage–n = 5 and non-carriage control–n = 20. (A) PCA scores showing the distribution of the three sample groups, S. Typhi carriage, S. Paratyphi A carriage, and non-carriage controls with an indication of separation of non-carriage controls from the Salmonella carriage samples. (B) OPLS-DA scores showing the separation of non-carriage controls from the Salmonella carriage samples along the first component and the separation of S. Typhi carriage samples from the S. Paratyphi A carriage samples along the second component (p = 0.0031). Additional model information is shown in Table 1. (TIF) [file pntd.0006215.s001.tif]

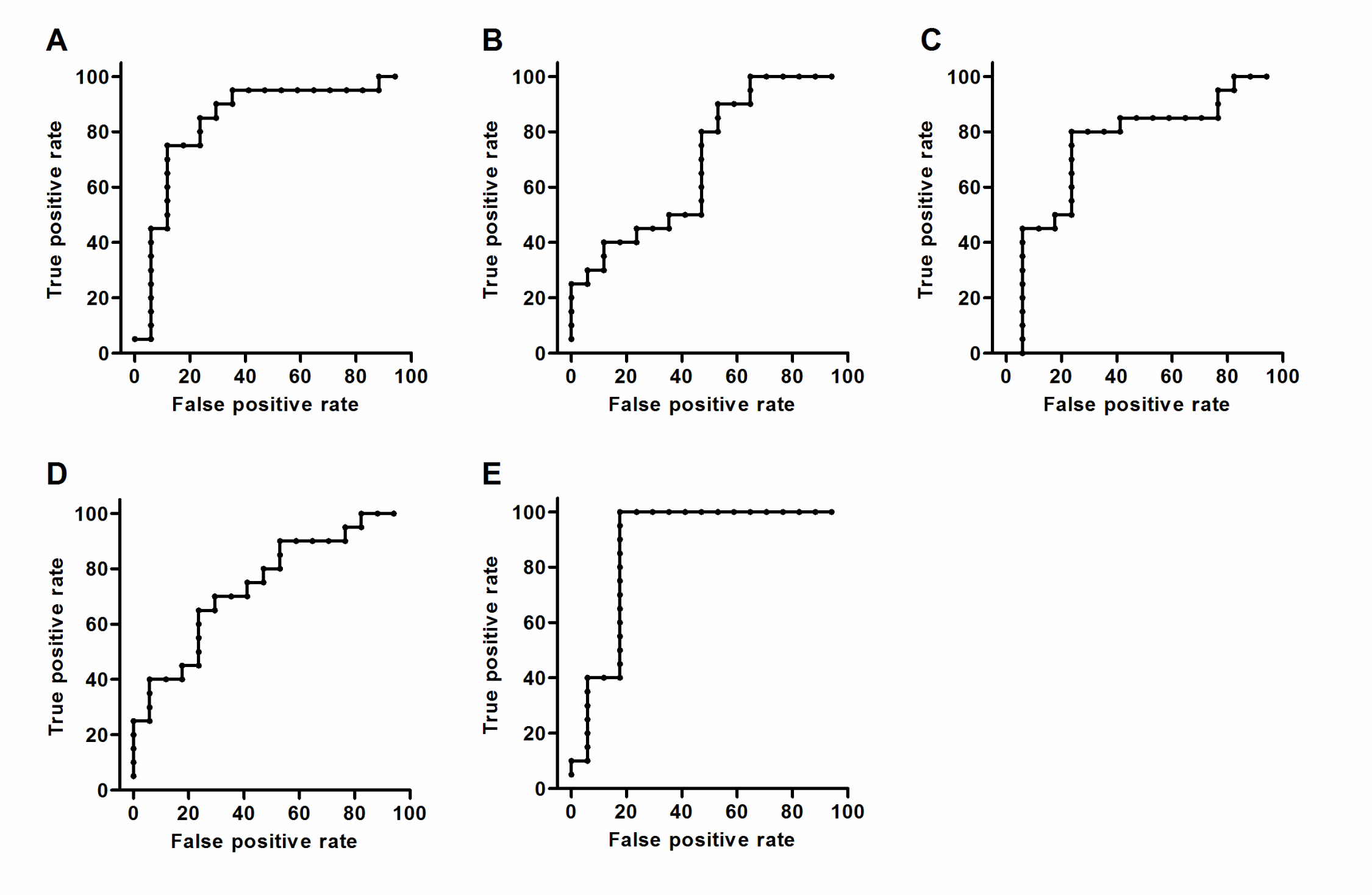

Supplement: S2 Fig — Panels A-E are showing ROC curves with false positive rates (i.e. 1-specificity) and true positive rates (i.e. sensitivity) on the x- and y-axes respectively. The ROC curves are constructed from relative metabolite concentrations comparing Salmonella carriage samples to non-carriage controls. AUC values are presented along with 95% confidence intervals. (A) Hexanoic acid (Caproic acid), AUC = 0.841 (0.680–0.968). (B) Unknown_087, AUC = 0.697 (0.521–0.864). (C) Unknown_118, AUC = 0.756 (0.589–0.904). (D) Glutaric acid, AUC = 0.774 (0.565–0.887). (E) Unknown_399, AUC = 0.876 (0.726–1). (TIF) [file pntd.0006215.s002.tif]
