# Supplementary material for: Diagnostic metabolite biomarkers of chronic typhoid carriage
Source: PLoS Negl Trop Dis. 2018 Jan 26;12(1):e0006215. doi: 10.1371/journal.pntd.0006215 (PMC5802941; doi:10.1371/journal.pntd.0006215)
Supplement: S1 Table — (DOCX) [file pntd.0006215.s004.docx]

**S1 Table. Patient group metadata.**

| **Parameter** | **Non-carriage controls** | | | ***S*. Typhi carriage** | | | ***S*. Paratyphi A carriage** | | |
| --- | --- | --- | --- | --- | --- | --- | --- | --- | --- |
| Quantitative parameter | n=20^a^ | Median | IQR^b^ | n=12^a^ | Median | IQR^b^ | n=5^a^ | Median | IQR^b^ |
| Qualitative parameter |  | Yes | %^c^ |  | Yes | %^c^ |  | Yes | %^c^ |
| **Clinical presentation and history** | | | | | | | | | |
| Age (Years) | 20 | 36 | 29-52 | 12 | 37 | (29-47) | 5 | 34 | 26-54 |
| Sex (Female) | 20 | 16 | 80 | 12 | 9 | 75 | 5 | 4 | 80 |
| Fever | 17 | 6 | 35.3 | 10 | 0 | 0 | 5 | 1 | 20 |
| Fever >5 days on entry | 16 | 3 | 18.8 | 11 | 1 | 9.1 | 5 | 1 | 20 |
| Previous history of typhoid | 3 | 2 | 66.7 | 1 | 1 | 100 | 2 | 1 | 50 |
| Culture confirmed typhoid | 2 | 0 | 0 | 0 | 0 | 0 | 2 | 0 | 0 |
| Typhoid vaccination | 18 | 0 | 0 | 11 | 0 | 0 | 4 | 0 | 0 |
| Antibiotics 15 days | 15 | 2 | 13.3 | 10 | 0 | 0 | 3 | 0 | 0 |
| Right upper quadrant pain | 18 | 15 | 83.3 | 12 | 9 | 75 | 5 | 4 | 80 |
| Right upper quadrant tenderness | 11 | 6 | 54.5 | 11 | 6 | 54.5 | 5 | 2 | 40 |
| Jaundice at admission | 17 | 0 | 0 | 12 | 1 | 8.3 | 5 | 1 | 20 |
| Previous jaundice | 19 | 4 | 21.1 | 12 | 2 | 16.7 | 4 | 1 | 25 |
| Previous hospital due to jaundice | 2 | 0 | 0 | 0 | 0 | 0 | 1 | 0 | 0 |
| Previous acute cholecystitis | 15 | 3 | 20 | 12 | 1 | 8.3 | 5 | 4 | 80 |
| Previous hospital due to cholecystitis | 3 | 3 | 100 | 1 | 1 | 100 | 4 | 1 | 25 |
| Pancreatitis | 16 | 1 | 6.3 | 12 | 1 | 8.3 | 4 | 0 | 0 |
| Diabetes | 19 | 1 | 5.3 | 12 | 1 | 8.3 | 5 | 0 | 0 |
| **Gallbladder and common bile duct parameters** | | | | | | | | | |
| Palpable gallbladder | 11 | 0 | 0 | 10 | 0 | 0 | 3 | 0 | 0 |
| Gallbladder tissue thickness |  |  |  |  |  |  |  |  |  |
| Thick | 13 | 1 | 7.7 | 6 | 1 | 16.7 | 3 | 1 | 33.3 |
| Thin | 13 | 3 | 23.1 | 6 | 5 | 83.3 | 3 | 0 | 0 |
| Gallbladder size |  |  |  |  |  |  |  |  |  |
| Distended | 8 | 8 | 100 | 3 | 3 | 100 | 2 | 1 | 50 |
| Contracted | 8 | 0 | 0 | 3 | 0 | 0 | 2 | 1 | 50 |
| Gall stones |  |  |  |  |  |  |  |  |  |
| Multiple | 19 | 10 | 52.6 | 11 | 9 | 81.8 | 5 | 3 | 60 |
| Single | 19 | 9 | 47.4 | 11 | 1 | 9.1 | 5 | 1 | 20 |
| Common bile duct stone |  |  |  |  |  |  |  |  |  |
| Single | 19 | 1 | 5.3 | 11 | 1 | 9.1 | 4 | 1 | 25 |
| Common bile duct diameter | 10 | 4 | 3-4.6 | 4 | 5.1 | 4.0-8.3 | 2 | 9.6 | - |
| Common bile duct diameter category |  |  |  |  |  |  |  |  |  |
| Dilated | 18 | 0 | 0 | 10 | 1 | 10 | 4 | 1 | 25 |
| Cholelithiasis | 15 | 1 | 6.7 | 8 | 8 | 100 | 2 | 2 | 100 |
| Surgery |  |  |  |  |  |  |  |  |  |
| Acute | 19 | 0 | 0 | 9 | 0 | 0 | 5 | 2 | 40 |
| Elective | 19 | 19 | 100 | 9 | 9 | 100 | 5 | 3 | 60 |
| Treatment with inj. Cefazoline | 10 | 10 | 100 | 3 | 3 | 100 | 1 | 1 | 100 |
| **Hematological and biochemical parameters** | | | | | | | | | |
| Total cell count | 17 | 7.2 | 6.4-9.2 | 12 | 9.4 | 6.5-17.3 | 5 | 11.7 | 6.5-13.2 |
| Neutrophils (%) | 17 | 67 | 62-76.5 | 12 | 69.5 | 60-83.3 | 5 | 76 | 0.5-84.5 |
| Lymphocytes (%) | 17 | 27 | 20.5-33 | 11 | 24 | 16-40 | 5 | 22 | 15.5-36 |
| Monocytes (%) | 8 | 1.5 | 1-3.8 | 2 | 3 | - | 1 | 1 | - |
| Eosinophils (%) | 14 | 2 | 1.8-3.3 | 3 | 2 | 1-5 | 3 | 2 | 2-4 |
| Total bilirubin (mg/mL) | 19 | 0.8 | 0.6-0.9 | 10 | 0.85 | 0.7-1.5 | 5 | 1.1 | 0.4-2.4 |
| Conjugated bilirubin (mg/mL) | 19 | 0.2 | 0.2-0.3 | 10 | 0.22 | 0.2-0.9 | 5 | 0.4 | 0.2-1 |
| Aspartate transaminase (u/L) | 19 | 32 | 24-41 | 11 | 35 | 22-43 | 5 | 25 | 24.5-67 |
| Alanine transaminase (u/L) | 19 | 32 | 26-44 | 11 | 39 | 32-72 | 5 | 28 | 24-74.5 |
| Alkaline phosphatase (u/L) | 18 | 75 | 57-94.8 | 11 | 173 | 82-300 | 5 | 85 | 66-853 |
| Amylase (u/L) | 4 | 73 | 50-1012 | 3 | 190 | 117-2300 | 2 | 32 | - |

^a^ Number of samples in each sample group in the top row followed by the number of available answers for each parameter.

^b^ IQR – Interquartile range.

^c^ Percentage of “Yes” answers or similar out of the number of answers for each parameter.
