# Supplementary material for: Diagnostic metabolite biomarkers of chronic typhoid carriage
Source: PLoS Negl Trop Dis. 2018 Jan 26;12(1):e0006215. doi: 10.1371/journal.pntd.0006215 (PMC5802941; doi:10.1371/journal.pntd.0006215)
Supplement: S2 Table — (DOCX) [file pntd.0006215.s005.docx]

**S2 Table. Detected metabolites in plasma samples of Salmonella carriers analyzed with GCxGC-TOFMS.**

| **Metabolite^a^** | **ID Info^b^** | **HMDB ID^c^** | **RT1^d^** | **RT2^d^** | **RI^d^** | **Significance^e^** | **Direction^f^** | **AUC (95% CI)^g^** | **p-value^h^** |
| --- | --- | --- | --- | --- | --- | --- | --- | --- | --- |
| 1,5-anhydro-D-glucitol | ID | HMDB02712 | 1950 | 2.93 | 1842 |  |  | 0.556 (0.357-0.735) | 0.57 |
| 1,6-anhydro-beta-d-glucose | ID |  | 1840 | 2.36 | 1767 |  |  | 0.535 (0.337-0.724) | 0.73 |
| 1-dodecanoyl-sn-glycero-3-phosphocholine | UC | HMDB62319 | 3515 | 2.61 | 3135 |  |  | 0.576 (0.385-0.754) | 0.44 |
| 1-monohexadecanoylglycerol | UC | HMDB31074 | 2975 | 2.18 | 2616 |  |  | 0.544 (0.347-0.738) | 0.66 |
| 1-monooleoylglycerol | UC |  | 3185 | 2.43 | 2797 |  |  | 0.512 (0.324-0.712) | 0.92 |
| 1-monostearoylglycerol | UC | HMDB31075 | 3185 | 2.46 | 2797 |  |  | 0.526 (0.327-0.719) | 0.80 |
| 2,3-dihydroxybutanoic acid | UC | HMDB00498 | 1120 | 3.09 | 1367 |  |  | 0.568 (0.365-0.765) | 0.49 |
| 2-aminobutyric acid | ID | HMDB00452 | 685 | 1.84 | 1136 |  |  | 0.529 (0.331-0.713) | 0.77 |
| 2-hydroxybutanoic acid | ID | HMDB00008 | 725 | 2.61 | 1157 |  |  | 0.544 (0.351-0.745) | 0.66 |
| 2-hydroxyglutaric acid | UC | HMDB00694 | 1610 | 2.55 | 1627 |  |  | 0.506 (0.303-0.695) | 0.96 |
| 2-hydroxypyridine | ID | HMDB13751 | 710 | 1.66 | 1150 | * | C | 0.732 (0.551-0.885) | 0.017 |
| 2-oxoisocaproic acid (Ketoleucine) | ID | HMDB00695 | 1060 | 2 | 1335 | * | C | 0.765 (0.601-0.906) | 0.0064 |
| 3,4-dihydroxybutanoic acid | ID | HMDB00337 | 1275 | 3.04 | 1449 |  |  | 0.518 (0.312-0.709) | 0.87 |
| 3-heptanone | ID | HMDB31482 | 495 | 1.58 | 1035 |  |  | 0.615 (0.425-0.809) | 0.24 |
| 3-hydroxybutyric acid | ID | HMDB00442 | 795 | 2.1 | 1195 |  |  | 0.568 (0.381-0.751) | 0.49 |
| 3-methyl-2-oxovaleric acid | UC | HMDB00491 | 975 | 1.84 | 1290 | * | C | 0.768 (0.598-0.906) | 0.0058 |
| Alanine | ID | HMDB00161 | 540 | 3.23 | 1059 |  |  | 0.559 (0.363-0.751) | 0.55 |
| Alpha-linolenic acid (Octadecantrienoic acid) | ID | HMDB01388 | 2650 | 2.44 | 2335 |  |  | 0.562 (0.37-0.76) | 0.53 |
| Aminomalonic acid | ID | HMDB01147 | 1440 | 2.25 | 1537 |  |  | 0.624 (0.43-0.804) | 0.21 |
| Arachidonic acid (Eicosatetraenoic acid) | ID | HMDB01043 | 2855 | 1.99 | 2512 |  |  | 0.647 (0.456-0.814) | 0.13 |
| Aspartic acid | ID |  | 1490 | 2.48 | 1563 |  |  | 0.565 (0.358-0.755) | 0.51 |
| Beta-alanine | ID | HMDB00056 | 1250 | 3.17 | 1436 |  |  | 0.597 (0.404-0.782) | 0.32 |
| Beta-sitosterol | UC | HMDB00852 | 3840 | 3.24 | 3475 |  |  | 0.521 (0.303-0.738) | 0.84 |
| Carbohydrate_513 | CL |  | 2160 | 3.34 | 1985 | * | C | 0.732 (0.547-0.88) | 0.017 |
| Chenodeoxycholic acid | ID | HMDB00518 | 3780 | 3.04 | 3412 |  |  | 0.547 (0.344-0.74) | 0.64 |
| Cholesterol | ID | HMDB00067 | 3660 | 2.9 | 3287 |  |  | 0.632 (0.444-0.818) | 0.18 |
| Citric acid | ID | HMDB00094 | 1965 | 2.36 | 1852 | * | C | 0.635 (0.439-0.807) | 0.17 |
| Creatinine | ID | HMDB00562 | 1600 | 2.51 | 1622 | * | C | 0.671 (0.501-0.826) | 0.080 |
| Cysteine | ID | HMDB00574 | 1570 | 2.4 | 1606 |  |  | 0.5 (0.297-0.703) | 1 |
| Decanoic acid (Capric acid) | ID | HMDB00511 | 1400 | 2.22 | 1516 | * | C | 0.709 (0.524-0.863) | 0.032 |
| Docosahexaenoic acid | ID | HMDB02183 | 3120 | 2.04 | 2741 |  |  | 0.588 (0.398-0.762) | 0.37 |
| Dodecanoic acid (Lauric acid) | ID | HMDB00638 | 1735 | 2.06 | 1696 |  |  | 0.629 (0.42-0.824) | 0.18 |
| Elaidic/Oleic acid (Octadecenoic acid) | ID | HMDB00573/HMDB00207 | 2590 | 2.67 | 2278 |  |  | 0.588 (0.391-0.773) | 0.37 |
| Erythritol/Threitol | ID | HMDB02994/HMDB04136 | 1285 | 3.79 | 1455 | * | C | 0.665 (0.473-0.835) | 0.091 |
| Ethanolamine | ID | HMDB00149 | 840 | 3.37 | 1218 | * | T/P | 0.641 (0.451-0.801) | 0.15 |
| Fatty_acid_612 | CL |  | 2830 | 2.07 | 2490 |  |  | 0.632 (0.441-0.811) | 0.18 |
| Fructose | ID | HMDB00660 | 1890 | 4.04 | 1801 |  |  | 0.55 (0.355-0.729) | 0.62 |
| Galacturonic acid | ID | HMDB02545 | 2035 | 3.43 | 1900 |  |  | 0.55 (0.353-0.742) | 0.62 |
| Glucose | ID | HMDB00122 | 1895 | 4.32 | 1805 |  |  | 0.682 (0.501-0.844) | 0.061 |
| Glutamic acid | ID | HMDB00148 | 1670 | 2.68 | 1659 |  |  | 0.579 (0.375-0.767) | 0.42 |
| Glutaric acid | ID | HMDB00661 | 1375 | 2.13 | 1502 | * | T/P | 0.744 (0.565-0.887) | 0.012 |
| Glycerol | ID | HMDB00131 | 910 | 3 | 1256 |  |  | 0.512 (0.333-0.713) | 0.92 |
| Glycerol-3-phosphate | ID | HMDB00126 | 1905 | 2.05 | 1812 | * | T/P | 0.718 (0.548-0.877) | 0.025 |
| Glycine | ID | HMDB00123 | 1015 | 3.19 | 1311 |  |  | 0.509 (0.299-0.703) | 0.94 |
| Glycolic acid | ID | HMDB00115 | 685 | 2.22 | 1136 | * | C | 0.709 (0.543-0.866) | 0.032 |
| Glyoxylic acid | UC | HMDB00119 | 655 | 1.74 | 1120 | * | C | 0.659 (0.474-0.838) | 0.10 |
| Hexanoic acid (Caproic acid) | ID | HMDB00535 | 680 | 2.13 | 1134 | * | T/P | 0.841 (0.68-0.968) | 0.00043 |
| Hydroxylamine | ID | HMDB03338 | 600 | 3.27 | 1091 |  |  | 0.579 (0.361-0.773) | 0.42 |
| Hydroxyproline | ID | HMDB00725 | 1440 | 3.12 | 1537 |  |  | 0.535 (0.338-0.724) | 0.73 |
| Indoleacetic acid | ID | HMDB00197 | 2510 | 1.53 | 2223 |  |  | 0.559 (0.366-0.76) | 0.55 |
| Isoleucine | ID | HMDB00172 | 1000 | 2.97 | 1303 |  |  | 0.506 (0.312-0.692) | 0.96 |
| Lactic acid | ID | HMDB00190 | 610 | 2.01 | 1096 |  |  | 0.559 (0.364-0.734) | 0.55 |
| Lactose | ID | HMDB00186 | 2860 | 3.86 | 2516 |  |  | 0.519 (0.307-0.714) | 0.86 |
| Leucine | ID | HMDB00687 | 960 | 3.08 | 1282 |  |  | 0.509 (0.324-0.706) | 0.94 |
| Linoleic acid (Octadecadienoic acid) | ID | HMDB00673 | 2615 | 2.57 | 2304 |  |  | 0.647 (0.465-0.824) | 0.13 |
| Lumichrome | UC |  | 1125 | 2.4 | 1370 |  |  | 0.615 (0.425-0.788) | 0.24 |
| Lysine | ID | HMDB00182 | 2010 | 3.4 | 1883 |  |  | 0.553 (0.367-0.746) | 0.59 |
| Malic acid | ID | HMDB00156 | 1440 | 2.71 | 1537 |  |  | 0.579 (0.374-0.772) | 0.43 |
| m-cresol | UC | HMDB02048 | 905 | 1.86 | 1253 |  |  | 0.565 (0.361-0.754) | 0.51 |
| Methionine | ID | HMDB00696 | 1570 | 2.29 | 1606 |  |  | 0.562 (0.367-0.749) | 0.53 |
| Methyl hexadecanoic acid | UC | HMDB61859 | 2255 | 2.01 | 2050 |  |  | 0.597 (0.393-0.783) | 0.32 |
| Monoethylhexyl phthalic acid | UC | HMDB13248 | 3220 | 1.79 | 2827 |  |  | 0.635 (0.444-0.805) | 0.17 |
| Monomethylphosphate | UC |  | 995 | 1.95 | 1301 |  |  | 0.556 (0.356-0.77) | 0.57 |
| Monosaccharide_412 | CL |  | 1785 | 3.17 | 1730 | * | C | 0.694 (0.51-0.861) | 0.050 |
| Monosaccharide_462 | CL |  | 1940 | 1.58 | 1835 | * | C | 0.732 (0.544-0.888) | 0.017 |
| Monosaccharide_463 | CL |  | 1940 | 1.66 | 1835 | * | C | 0.618 (0.424-0.802) | 0.23 |
| Monosaccharide_487 | CL |  | 2060 | 3.58 | 1917 | * | C | 0.731 (0.549-0.879) | 0.019 |
| Myo-inositol | ID | HMDB00211 | 2125 | 0.02 | 1961 |  |  | 0.641 (0.432-0.816) | 0.15 |
| Myo-inositol-1-phosphate | ID | HMDB00213 | 2630 | 3.43 | 2317 |  |  | 0.556 (0.363-0.741) | 0.57 |
| Myo-inositol-2-phosphate | UC |  | 2715 | 3.55 | 2391 |  |  | 0.55 (0.344-0.744) | 0.62 |
| Nonanoic acid | ID | HMDB00847 | 1220 | 2.39 | 1420 | * | C | 0.879 (0.729-1) | 0.000090 |
| Octanoic acid (Caprylic acid) | ID | HMDB00482 | 1035 | 2.01 | 1322 | * | C | 0.897 (0.764-0.994) | 0.000041 |
| Ornithine | ID | HMDB00214 | 1850 | 3.54 | 1774 |  |  | 0.562 (0.351-0.745) | 0.53 |
| Pentadecanoic acid | ID | HMDB00826 | 2190 | 2.5 | 2005 |  |  | 0.706 (0.529-0.87) | 0.034 |
| Phenylalanine | ID | HMDB00159 | 1770 | 2.23 | 1720 |  |  | 0.55 (0.363-0.745) | 0.62 |
| Phosphoric acid | ID | HMDB02142 | 1070 | 2.54 | 1341 |  |  | 0.506 (0.323-0.693) | 0.96 |
| Pipecolic acid | UC | HMDB00070 | 1120 | 2.47 | 1367 |  |  | 0.585 (0.395-0.768) | 0.39 |
| Pseudouridine | UC | HMDB00767 | 2705 | 2.03 | 2382 | * | C | 0.8 (0.63-0.939) | 0.0020 |
| Pyroglutamic acid | ID | HMDB00267 | 1705 | 2.13 | 1676 |  |  | 0.55 (0.339-0.754) | 0.62 |
| Pyruvic acid | ID | HMDB00243 | 770 | 1.5 | 1181 |  |  | 0.618 (0.418-0.803) | 0.23 |
| Ribitol (or isomer) | ID | HMDB00508 | 1610 | 3.76 | 1627 | * | C | 0.668 (0.473-0.835) | 0.085 |
| Saccharide_421 | CL |  | 1810 | 3.59 | 1747 |  |  | 0.603 (0.406-0.799) | 0.29 |
| Serine | ID | HMDB00187 | 1060 | 2.07 | 1335 |  |  | 0.503 (0.312-0.693) | 0.99 |
| S-methylcysteine | ID | HMDB02108 | 1390 | 2.09 | 1510 |  |  | 0.541 (0.342-0.733) | 0.68 |
| Stearic acid (Octadecanoic acid) | ID | HMDB00827 | 2590 | 2.79 | 2278 |  |  | 0.644 (0.459-0.812) | 0.14 |
| Sucrose | ID | HMDB00258 | 2825 | 4.08 | 2486 |  |  | 0.515 (0.299-0.724) | 0.89 |
| Sugar_alcohol_446 | CL |  | 1880 | 0.13 | 1795 |  |  | 0.641 (0.44-0.822) | 0.15 |
| Threonic/Erythronic acid | ID | HMDB00943/HMDB00613 | 1480 | 2.4 | 1558 |  |  | 0.524 (0.329-0.731) | 0.82 |
| Threonine | ID | HMDB00167 | 1155 | 3.35 | 1386 |  |  | 0.509 (0.322-0.718) | 0.94 |
| Tryptophan | ID | HMDB30396 | 2720 | 2.25 | 2395 | * | C | 0.553 (0.344-0.738) | 0.59 |
| Tyrosine | ID | HMDB00158 | 2275 | 1.99 | 2063 |  |  | 0.526 (0.335-0.721) | 0.80 |
| Unknown_013 | UN |  | 540 | 1.85 | 1059 |  |  | 0.568 (0.365-0.753) | 0.49 |
| Unknown_027 | UN |  | 550 | 3.1 | 1065 | * | T/P | 0.715 (0.546-0.873) | 0.027 |
| Unknown_032 | UN |  | 570 | 3.26 | 1075 |  |  | 0.668 (0.473-0.856) | 0.085 |
| Unknown_034 | UN |  | 575 | 3.28 | 1078 |  |  | 0.585 (0.389-0.778) | 0.39 |
| Unknown_035 | UN |  | 580 | 3.43 | 1081 |  |  | 0.568 (0.372-0.765) | 0.49 |
| Unknown_046 | UN |  | 610 | 1.57 | 1096 |  |  | 0.635 (0.456-0.814) | 0.17 |
| Unknown_049 | UN |  | 615 | 3.52 | 1099 |  |  | 0.665 (0.491-0.84) | 0.091 |
| Unknown_064 | UN |  | 665 | 3.22 | 1126 |  |  | 0.529 (0.323-0.729) | 0.77 |
| Unknown_067 | UN |  | 670 | 3.19 | 1128 |  |  | 0.55 (0.356-0.741) | 0.62 |
| Unknown_079 | UN |  | 705 | 2.51 | 1147 |  |  | 0.503 (0.308-0.706) | 0.99 |
| Unknown_082 | UN |  | 720 | 1.97 | 1155 |  |  | 0.553 (0.353-0.741) | 0.59 |
| Unknown_087 | UN |  | 730 | 2.15 | 1160 | * | T/P | 0.697 (0.521-0.864) | 0.043 |
| Unknown_091 | UN |  | 745 | 2.57 | 1168 |  |  | 0.582 (0.358-0.774) | 0.40 |
| Unknown_102 | UN |  | 790 | 3.06 | 1192 | * | C | 0.816 (0.647-0.944) | 0.0014 |
| Unknown_105 | UN |  | 825 | 2.99 | 1211 |  |  | 0.591 (0.397-0.793) | 0.35 |
| Unknown_106 | UN |  | 825 | 3.15 | 1211 |  |  | 0.603 (0.398-0.799) | 0.29 |
| Unknown_118 | UN |  | 840 | 2.21 | 1218 | * | T/P | 0.756 (0.589-0.904) | 0.0084 |
| Unknown_119 | UN |  | 840 | 2.3 | 1218 |  |  | 0.574 (0.378-0.753) | 0.46 |
| Unknown_134 | UN |  | 895 | 3.13 | 1248 |  |  | 0.521 (0.333-0.717) | 0.84 |
| Unknown_160 | UN |  | 970 | 2.18 | 1287 | * | C | 0.665 (0.486-0.83) | 0.091 |
| Unknown_164 | UN |  | 980 | 1.71 | 1293 |  |  | 0.606 (0.409-0.798) | 0.28 |
| Unknown_168 | UN |  | 995 | 2.13 | 1301 |  |  | 0.509 (0.302-0.698) | 0.94 |
| Unknown_169 | UN |  | 995 | 2.14 | 1301 |  |  | 0.565 (0.369-0.754) | 0.51 |
| Unknown_186 | UN |  | 1020 | 1.74 | 1314 |  |  | 0.506 (0.319-0.707) | 0.96 |
| Unknown_192 | UN |  | 1045 | 1.58 | 1327 |  |  | 0.574 (0.375-0.77) | 0.46 |
| Unknown_241 | UN |  | 1200 | 1.63 | 1409 |  |  | 0.565 (0.363-0.74) | 0.51 |
| Unknown_245 | UN |  | 1225 | 3.82 | 1423 | * | C | 0.668 (0.488-0.826) | 0.085 |
| Unknown_246 | UN |  | 1225 | 2.51 | 1423 | * | C | 0.753 (0.586-0.898) | 0.0092 |
| Unknown_249 | UN |  | 1250 | 3.64 | 1436 |  |  | 0.506 (0.308-0.696) | 0.96 |
| Unknown_252 | UN |  | 1260 | 2.42 | 1441 |  |  | 0.6 (0.408-0.788) | 0.31 |
| Unknown_261 | UN |  | 1300 | 3.2 | 1463 |  |  | 0.626 (0.429-0.813) | 0.20 |
| Unknown_262 | UN |  | 1300 | 3.62 | 1463 |  |  | 0.629 (0.449-0.81) | 0.18 |
| Unknown_265 | UN |  | 1310 | 1.87 | 1468 |  |  | 0.576 (0.382-0.756) | 0.44 |
| Unknown_273 | UN |  | 1360 | 1.6 | 1494 |  |  | 0.665 (0.482-0.841) | 0.091 |
| Unknown_279 | UN |  | 1385 | 2.07 | 1508 | * | C | 0.676 (0.488-0.848) | 0.070 |
| Unknown_281 | UN |  | 1385 | 2.58 | 1508 |  |  | 0.647 (0.454-0.814) | 0.13 |
| Unknown_288 | UN |  | 1390 | 3.38 | 1510 | * | C | 0.712 (0.538-0.87) | 0.029 |
| Unknown_290 | UN |  | 1395 | 2.08 | 1513 |  |  | 0.515 (0.316-0.705) | 0.89 |
| Unknown_306 | UN |  | 1440 | 1.47 | 1537 |  |  | 0.574 (0.379-0.752) | 0.46 |
| Unknown_315 | UN |  | 1475 | 1.69 | 1555 | * | C | 0.679 (0.491-0.841) | 0.065 |
| Unknown_319 | UN |  | 1485 | 2.17 | 1561 |  |  | 0.568 (0.36-0.753) | 0.49 |
| Unknown_328 | UN |  | 1500 | 1.95 | 1569 | * | C | 0.868 (0.728-0.963) | 0.00015 |
| Unknown_340 | UN |  | 1535 | 1.8 | 1587 | * | C | 0.829 (0.68-0.945) | 0.00068 |
| Unknown_341 | UN |  | 1540 | 2.5 | 1590 | * | C | 0.688 (0.494-0.85) | 0.053 |
| Unknown_345 | UN |  | 1560 | 1.65 | 1600 |  |  | 0.535 (0.327-0.717) | 0.73 |
| Unknown_352 | UN |  | 1595 | 2.48 | 1619 | * | C | 0.871 (0.712-0.98) | 0.00013 |
| Unknown_368 | UN |  | 1645 | 2.26 | 1646 | * | C | 0.626 (0.442-0.807) | 0.20 |
| Unknown_370 | UN |  | 1655 | 1.36 | 1651 |  |  | 0.529 (0.331-0.716) | 0.77 |
| Unknown_373 | UN |  | 1660 | 2.21 | 1654 |  |  | 0.521 (0.328-0.721) | 0.84 |
| Unknown_375 | UN |  | 1660 | 3.59 | 1654 |  |  | 0.612 (0.421-0.795) | 0.25 |
| Unknown_384 | UN |  | 1695 | 2.42 | 1672 | * | C | 0.709 (0.523-0.865) | 0.034 |
| Unknown_393 | UN |  | 1730 | 3.17 | 1693 |  |  | 0.556 (0.363-0.737) | 0.57 |
| Unknown_395 | UN |  | 1755 | 2 | 1710 | * | C | 0.722 (0.546-0.881) | 0.025 |
| Unknown_397 | UN |  | 1760 | 2.03 | 1713 |  |  | 0.609 (0.408-0.791) | 0.27 |
| Unknown_399 | UN |  | 1755 | 2.25 | 1710 | * | T/P | 0.876 (0.726-1) | 0.00010 |
| Unknown_400 | UN |  | 1760 | 2.27 | 1713 |  |  | 0.624 (0.425-0.802) | 0.21 |
| Unknown_419 | UN |  | 1810 | 2.08 | 1747 |  |  | 0.594 (0.393-0.765) | 0.34 |
| Unknown_420 | UN |  | 1810 | 2.09 | 1747 |  |  | 0.521 (0.318-0.725) | 0.84 |
| Unknown_424 | UN |  | 1820 | 3.52 | 1754 |  |  | 0.729 (0.541-0.889) | 0.018 |
| Unknown_425 | UN |  | 1835 | 1.26 | 1764 |  |  | 0.503 (0.295-0.689) | 0.99 |
| Unknown_428 | UN |  | 1835 | 3.14 | 1764 |  |  | 0.624 (0.417-0.795) | 0.21 |
| Unknown_435 | UN |  | 1845 | 3.27 | 1771 | * | C | 0.671 (0.488-0.821) | 0.080 |
| Unknown_445 | UN |  | 1875 | 3.41 | 1791 | * | C | 0.741 (0.563-0.893) | 0.015 |
| Unknown_470 | UN |  | 1990 | 3.4 | 1869 | * | C | 0.847 (0.705-0.96) | 0.00044 |
| Unknown_471 | UN |  | 2000 | 3.15 | 1876 | * | C | 0.75 (0.58-0.894) | 0.010 |
| Unknown_483 | UN |  | 2045 | 1.59 | 1907 |  |  | 0.585 (0.385-0.767) | 0.39 |
| Unknown_485 | UN |  | 2050 | 2.99 | 1910 |  |  | 0.579 (0.386-0.761) | 0.42 |
| Unknown_489 | UN |  | 2080 | 1.6 | 1931 | * | C | 0.865 (0.725-0.961) | 0.00017 |
| Unknown_494 | UN |  | 2095 | 3.03 | 1941 |  |  | 0.576 (0.37-0.78) | 0.44 |
| Unknown_498 | UN |  | 2120 | 1.69 | 1958 | * | C | 0.782 (0.612-0.917) | 0.0036 |
| Unknown_501 | UN |  | 2135 | 2.21 | 1968 | * | C | 0.912 (0.804-0.985) | 0.000021 |
| Unknown_505 | UN |  | 2140 | 2.81 | 1971 |  |  | 0.518 (0.315-0.716) | 0.87 |
| Unknown_509 | UN |  | 2150 | 3.12 | 1978 | * | C | 0.774 (0.596-0.914) | 0.0048 |
| Unknown_510 | UN |  | 2150 | 3.15 | 1978 | * | C | 0.759 (0.584-0.903) | 0.0077 |
| Unknown_523 | UN |  | 2250 | 1.83 | 2046 |  |  | 0.556 (0.36-0.734) | 0.57 |
| Unknown_525 | UN |  | 2255 | 2.19 | 2050 | * | C | 0.835 (0.69-0.949) | 0.00054 |
| Unknown_545 | UN |  | 2390 | 3.51 | 2142 |  |  | 0.621 (0.43-0.791) | 0.22 |
| Unknown_547 | UN |  | 2405 | 3.07 | 2152 | * | C | 0.868 (0.726-0.97) | 0.00015 |
| Unknown_556 | UN |  | 2465 | 3.61 | 2193 |  |  | 0.615 (0.434-0.783) | 0.24 |
| Unknown_557 | UN |  | 2475 | 3.45 | 2199 |  |  | 0.556 (0.354-0.737) | 0.57 |
| Unknown_565 | UN |  | 2515 | 3.33 | 2227 |  |  | 0.538 (0.319-0.719) | 0.70 |
| Unknown_574 | UN |  | 2535 | 2.98 | 2240 |  |  | 0.529 (0.339-0.72) | 0.77 |
| Unknown_591 | UN |  | 2650 | 2.23 | 2335 | * | C | 0.803 (0.646-0.94) | 0.0018 |
| Unknown_593 | UN |  | 2685 | 1.99 | 2365 | * | C | 0.774 (0.594-0.912) | 0.0048 |
| Unknown_594 | UN |  | 2685 | 2.52 | 2365 | * | C | 0.806 (0.655-0.941) | 0.0016 |
| Unknown_603 | UN |  | 2720 | 1.88 | 2395 | * | C | 0.809 (0.652-0.939) | 0.0014 |
| Unknown_609 | UN |  | 2800 | 1.87 | 2464 | * | C | 0.788 (0.631-0.923) | 0.0030 |
| Unknown_611 | UN |  | 2805 | 3.94 | 2469 |  |  | 0.65 (0.455-0.827) | 0.12 |
| Unknown_618 | UN |  | 2845 | 2 | 2503 |  |  | 0.603 (0.413-0.783) | 0.29 |
| Unknown_623 | UN |  | 2860 | 1.98 | 2516 | * | C | 0.794 (0.626-0.93) | 0.0024 |
| Unknown_627 | UN |  | 2895 | 2.46 | 2547 | * | C | 0.71 (0.518-0.872) | 0.030 |
| Unknown_631 | UN |  | 2905 | 0.94 | 2555 |  |  | 0.509 (0.321-0.695) | 0.94 |
| Unknown_633 | UN |  | 2945 | 1.72 | 2590 | * | C | 0.674 (0.476-0.842) | 0.075 |
| Unknown_635 | UN |  | 2970 | 3.21 | 2612 | * | C | 0.662 (0.471-0.833) | 0.10 |
| Unknown_643 | UN |  | 3065 | 3.06 | 2694 |  |  | 0.579 (0.38-0.75) | 0.42 |
| Unknown_646 | UN |  | 3090 | 3.78 | 2715 |  |  | 0.571 (0.368-0.764) | 0.47 |
| Unknown_666 | UN |  | 3575 | 1.31 | 3198 |  |  | 0.603 (0.406-0.789) | 0.29 |
| Unknown_679 | UN |  | 3660 | 1.45 | 3287 |  |  | 0.541 (0.35-0.72) | 0.68 |
| Unknown_682 | UN |  | 3725 | 2.59 | 3355 |  |  | 0.571 (0.382-0.766) | 0.47 |
| Unknown_691 | UN |  | 3970 | 3.48 | 3610 |  |  | 0.574 (0.354-0.773) | 0.46 |
| Urea | ID | HMDB00294 | 1150 | 2.18 | 1383 |  |  | 0.526 (0.327-0.73) | 0.80 |
| Uric acid | ID | HMDB00289 | 2550 | 2.29 | 2250 | * | C | 0.735 (0.579-0.884) | 0.015 |
| Valine | ID | HMDB34366 | 855 | 3.04 | 1226 |  |  | 0.506 (0.322-0.706) | 0.96 |
| Xylulose | UC | HMDB01644 | 1660 | 3.32 | 1654 |  |  | 0.553 (0.35-0.746) | 0.59 |

^a^ Metabolites: Metabolite name where name within parenthesis refers to another synonym and name after the slash refers to another isomer or other cases where complete distinction between the metabolites is not possible with the used technique.

^b^ ID Info refers to level of identification; ID: putatively annotated metabolite, CL: assigned metabolite class, UC: uncertain identity, UN: unknown identity

^c^ HMDB ID; ID number from the Human Metabolome Database

^d^ RT1 refers to the 1^st^ dimension retention time (s), RT2 refers to the 2^nd^ dimension retention time (s), RI1 refers to the 1^st^ dimension retention index.

^e^ Significance: Metabolites with latent significance are denoted with *.

^f^ Direction refers to direction of change in relative metabolite concentration in the OPLS-DA model comparing *Salmonella* carriage samples and non-carriage controls, where metabolites with higher relative concentration in the non-carriage controls are marked with C and metabolites with higher relative concentration in the S. Typhi/S. Paratyphi A carriage samples are marked with T/P.

^g^ AUC (95% CI): Area under the curve values for receiver operating characteristic (ROC) curves based on relative metabolite concentrations comparing the *Salmonella* carriage samples to the non-carriage controls. AUC values ranging between 0.5 and 1. Including 95% confidence interval for the AUC values calculated using the bootstrap percentile resampling methods (using 1000 bootstrappings).

^h^ p-value: Univariate p-value resulting from the Mann-Whitney U-test.
