# Supplementary material for: Diagnostic metabolite biomarkers of chronic typhoid carriage
Source: PLoS Negl Trop Dis. 2018 Jan 26;12(1):e0006215. doi: 10.1371/journal.pntd.0006215 (PMC5802941; doi:10.1371/journal.pntd.0006215)
Supplement: S3 Table — (DOCX) [file pntd.0006215.s006.docx]

**S3 Table.** **Comparison of metabolites between acute enteric fever and chronic carriage.**

| **Metabolite^a^** | **ID Info^b^** | **HMDB ID^c^** | **Significance Chronic^d^** | **Significance Acute^d^** | **Direction Chronic^e^** | **Direction Acute^e^** |
| --- | --- | --- | --- | --- | --- | --- |
| **Same direction of change in chronic and acute samples** | | | | | | |
| Ethanolamine | ID | HMDB00149 | * | *† | T/P | T/P |
| Glycerol-3-phosphate | ID | HMDB00126 | *‡ | *† | T/P | T/P |
| Unknown_027 | UN |  | *‡ | *† | T/P | T/P |
| Ribitol (or isomer) | ID | HMDB00508 | * | *† | C | C |
| Tryptophan | ID | HMDB30396 | * | *† | C | C |
| Unknown_245 | UN |  | * | *† | C | C |
| Unknown_341 | UN |  | * | *† | C | C |
| Unknown_435 | UN |  | * | *† | C | C |
| Unknown_471 | UN |  | *‡ | *† | C | C |
| Unknown_509 | UN |  | *‡ | *† | C | C |
| **Different direction of change in chronic and acute samples** | | | | | | |
| 2-oxoisocaproic acid (Ketoleucine) | ID | HMDB00695 | *‡ | *† | C | T/P |
| 3-methyl-2-oxovaleric acid | UC | HMDB00491 | *‡ | *† | C | T/P |
| Creatinine | ID | HMDB00562 | * | *† | C | T/P |
| Decanoic acid (Capric acid) | ID | HMDB00511 | *‡ | *† | C | T/P |
| Erythritol/Threitol | ID | HMDB02994/HMDB04136 | * | *† | C | T/P |
| Monosaccharide_412 | CL |  | * | *† | C | T/P |
| Octanoic acid (Caprylic acid) | ID | HMDB00482 | *‡ | *† | C | T/P |
| Pseudouridine | UC | HMDB00767 | *‡ | *† | C | T/P |
| Unknown_384 | UN |  | *‡ | *† | C | T/P |
| Unknown_470 | UN |  | *‡ | *† | C | T/P |
| Unknown_498 | UN |  | *‡ | *† | C | T/P |
| Unknown_591 | UN |  | *‡ | *† | C | T/P |
| Unknown_594 | UN |  | *‡ | *† | C | T/P |
| Unknown_609 | UN |  | *‡ | *† | C | T/P |
| Unknown_623 | UN |  | *‡ | *† | C | T/P |
| Unknown_635 | UN |  | * | *† | C | T/P |
| **Only significant in the chronic samples** | | | | | | |
| Glutaric acid | ID | HMDB00661 | *‡ | n.s. | T/P |  |
| Hexanoic acid (Caproic acid) | ID | HMDB00535 | *‡ | n.s. | T/P |  |
| Unknown_087 | UN |  | *‡ | -  - | T/P |  |
| Unknown_118 | UN |  | *‡ | - | T/P |  |
| Unknown_399 | UN |  | *‡ | - | T/P |  |
| 2-hydroxypyridine | ID | HMDB13751 | *‡ | n.s. | C |  |
| Carbohydrate_513 | CL |  | *‡ | n.s. | C |  |
| Citric acid | ID | HMDB00094 | * | n.s. | C |  |
| Glycolic acid | ID | HMDB00115 | *‡ | n.s. | C |  |
| Monosaccharide_487 | CL |  | *‡ | n.s. | C |  |
| Nonanoic acid | ID | HMDB00847 | *‡ | n.s. | C |  |
| Unknown_102 | UN |  | *‡ | n.s. | C |  |
| Unknown_160 | UN |  | * | n.s. | C |  |
| Unknown_315 | UN |  | * | n.s. | C |  |
| Unknown_328 | UN |  | *‡ | n.s. | C |  |
| Unknown_340 | UN |  | *‡ | n.s. | C |  |
| Unknown_352 | UN |  | *‡ | n.s. | C |  |
| Unknown_368 | UN |  | * | n.s. | C |  |
| Unknown_395 | UN |  | *‡ | n.s. | C |  |
| Unknown_445 | UN |  | *‡ | n.s. | C |  |
| Unknown_489 | UN |  | *‡ | n.s. | C |  |
| Unknown_501 | UN |  | *‡ | n.s. | C |  |
| Unknown_627 | UN |  | *‡ | n.s. | C |  |
| Uric acid | ID | HMDB00289 | *‡ | n.s. | C |  |
| Glyoxylic acid | UC | HMDB00119 | * | - | C |  |
| Monosaccharide_462 | CL |  | *‡ | - | C |  |
| Monosaccharide_463 | CL |  | * | - | C |  |
| Unknown_246 | UN |  | *‡ | - | C |  |
| Unknown_279 | UN |  | * | - | C |  |
| Unknown_288 | UN |  | *‡ | - | C |  |
| Unknown_510 | UN |  | *‡ | - | C |  |
| Unknown_525 | UN |  | *‡ | - | C |  |
| Unknown_547 | UN |  | *‡ | - | C |  |
| Unknown_593 | UN |  | *‡ | - | C |  |
| Unknown_603 | UN |  | *‡ | - | C |  |
| Unknown_633 | UN |  | * | - | C |  |
| **Only significant in the acute samples** | | | | | | |
| 1-monohexadecanoylglycerol | UC | HMDB31074 | n.s. | *† |  | T/P |
| 1-monostearoylglycerol | UC | HMDB31075 | n.s. | *† |  | T/P |
| 2-aminobutyric acid | ID | HMDB00452 | n.s. | *† |  | T/P |
| 2-hydroxybutanoic acid | ID | HMDB00008 | n.s. | *† |  | T/P |
| 3,4-dihydroxybutanoic acid | ID | HMDB00337 | n.s. | *† |  | T/P |
| 3-hydroxybutyric acid | ID | HMDB00442 | n.s. | *† |  | T/P |
| Beta-alanine | ID | HMDB00056 | n.s. | *† |  | T/P |
| Docosahexaenoic acid | ID | HMDB02183 | n.s. | *† |  | T/P |
| Dodecanoic acid (Lauric acid) | ID | HMDB00638 | n.s. | *† |  | T/P |
| Elaidic/Oleic acid (Octadecenoic acid) | ID | HMDB00573/HMDB00207 | n.s. | *† |  | T/P |
| Glycerol | ID | HMDB00131 | n.s. | *† |  | T/P |
| Hydroxylamine | ID | HMDB03338 | n.s. | *† |  | T/P |
| Isoleucine | ID | HMDB00172 | n.s. | *† |  | T/P |
| Leucine | ID | HMDB00687 | n.s. | *† |  | T/P |
| Malic acid | ID | HMDB00156 | n.s. | *† |  | T/P |
| Phenylalanine | ID | HMDB00159 | n.s. | *† |  | T/P |
| Phosphoric acid | ID | HMDB02142 | n.s. | *† |  | T/P |
| Pipecolic acid | UC | HMDB00070 | n.s. | *† |  | T/P |
| Pyroglutamic acid | ID | HMDB00267 | n.s. | *† |  | T/P |
| Pyruvic acid | ID | HMDB00243 | n.s. | *† |  | T/P |
| Serine | ID | HMDB00187 | n.s. | *† |  | T/P |
| Stearic acid (Octadecanoic acid) | ID | HMDB00827 | n.s. | *† |  | T/P |
| Unknown_064 | UN |  | n.s. | *† |  | T/P |
| Unknown_091 | UN |  | n.s. | *† |  | T/P |
| Unknown_119 | UN |  | n.s. | *† |  | T/P |
| Unknown_169 | UN |  | n.s. | *† |  | T/P |
| Unknown_241 | UN |  | n.s. | *† |  | T/P |
| Unknown_262 | UN |  | n.s. | *† |  | T/P |
| Unknown_373 | UN |  | n.s. | *† |  | T/P |
| Unknown_400 | UN |  | n.s. | *† |  | T/P |
| Urea | ID | HMDB00294 | n.s. | *† |  | T/P |
| Valine | ID | HMDB34366 | n.s. | *† |  | T/P |
| Aminomalonic acid | ID | HMDB01147 | n.s. | *† |  | C |
| Cholesterol | ID | HMDB00067 | n.s. | *† |  | C |
| Fructose | ID | HMDB00660 | n.s. | *† |  | C |
| Galacturonic acid | ID | HMDB02545 | n.s. | *† |  | C |
| Lysine | ID | HMDB00182 | n.s. | *† |  | C |
| Methyl hexadecanoic acid | UC | HMDB61859 | n.s. | *† |  | C |
| Myo-inositol-1-phosphate | ID | HMDB00213 | n.s. | *† |  | C |
| S-methylcysteine | ID | HMDB02108 | n.s. | *† |  | C |
| Threonic/Erythronic acid | ID | HMDB00943/HMDB00613 | n.s. | *† |  | C |
| Tyrosine | ID | HMDB00158 | n.s. | *† |  | C |
| Unknown_393 | UN |  | n.s. | *† |  | C |
| Unknown_485 | UN |  | n.s. | *† |  | C |
| Unknown_494 | UN |  | n.s. | *† |  | C |
| Unknown_523 | UN |  | n.s. | *† |  | C |
| Unknown_556 | UN |  | n.s. | *† |  | C |
| Unknown_557 | UN |  | n.s. | *† |  | C |
| Unknown_565 | UN |  | n.s. | *† |  | C |
| Unknown_574 | UN |  | n.s. | *† |  | C |
| Unknown_611 | UN |  | n.s. | *† |  | C |
| Unknown_691 | UN |  | n.s. | *† |  | C |

^a^ Metabolite: Metabolite name where name within parenthesis refers to another synonym and name after the slash refers to another isomer or other cases where complete distinction between the metabolites is not possible with the used technique.

^b^ ID Info refers to level of identification; ID: putatively annotated metabolite, CL: assigned metabolite class, UC: uncertain identity, UN: unknown identity

^c^ HMDB ID; ID number from the Human Metabolome Database

^d^ Significance refers to multivariate significance where metabolites with latent significance are marked with * and univariate significance where metabolites with *p*≤0.05 in a Student´s t-test are marked with † and metabolites with *p*≤0.05 in a Mann-Whitney U-test are marked with ‡. Metabolites that are not significant are marked with n.s. and metabolites that are not present are marked with -.

^e^ Direction refers to direction of change in relative metabolite concentration in OPLS-DA models comparing *Salmonella* carriage samples vs. non-carriage controls in the chronic stage and acute *Salmonella* infection vs. afebrile controls in the acute stage. Metabolites with higher relative concentration in the controls are marked with C and metabolites with higher relative concentration in the *S*. Typhi/*S*. Paratyphi A group are marked with T/P.
